# Ice core evidence for atmospheric oxygen decline since the Mid-Pleistocene transition

**DOI:** 10.1126/sciadv.abj9341

**Published:** 2021-12-15

**Authors:** Yuzhen Yan, Edward J. Brook, Andrei V. Kurbatov, Jeffrey P. Severinghaus, John A. Higgins

**Affiliations:** 1 Department of Geosciences, Princeton University, Princeton, NJ, USA.; 2 Department of Earth, Environmental and Planetary Sciences, Rice University, Houston, TX, USA.; 3 College of Earth, Ocean, and Atmospheric Sciences, Oregon State University, Corvallis, OR, USA.; 4 Climate Change Institute, University of Maine, Orono, ME, USA.; 5 Scripps Institution of Oceanography, University of California, San Diego, La Jolla, CA, USA.

## Abstract

The history of atmospheric oxygen (*P*O_2_) and the processes that act to regulate it remain enigmatic because of difficulties in quantitative reconstructions using indirect proxies. Here, we extend the ice-core record of *P*O_2_ using 1.5-million-year-old (Ma) discontinuous ice samples drilled from Allan Hills Blue Ice Area, East Antarctica. No statistically significant difference exists in *P*O_2_ between samples at 1.5 Ma and 810 thousand years (ka), suggesting that the Late-Pleistocene imbalance in O_2_ sources and sinks began around the time of the transition from 40- to 100-ka glacial cycles in the Mid-Pleistocene between ~1.2 Ma and 700 ka. The absence of a coeval secular increase in atmospheric CO_2_ over the past ~1 Ma requires negative feedback mechanisms such as *P*co_2_-dependent silicate weathering. Fast processes must also act to suppress the immediate *P*co_2_ increase because of the imbalance in O_2_ sinks over sources beginning in the Mid-Pleistocene.

## Teaser

The 1.5-million-year-old Antarctic ice indicates a balanced atmospheric oxygen budget before the Mid-Pleistocene Transition.

## INTRODUCTION

Oxygen (O_2_) is an important life-supporting chemical species that plays a profound role in biological and geochemical processes on Earth. Past variations of O_2_ partial pressure (*P*O_2_) are commonly derived from geochemical models constrained by carbon and/or sulfur isotopes (*1*–*3*) and from proxies that are sensitive to the presence of oxygen, such as multiple sulfur isotopes (*4*), isotopes of redox-sensitive metals (*5*), iodine speciation (*6*), and the abundance of charcoal (*7*). The magnitude of the modeled or proxy-based *P*O_2_ variations, however, often disagrees in both magnitude and sign (*8*), calling for more accurate reconstructions of the atmospheric O_2_ concentration. Attempts to directly measure past *P*O_2_ were limited to a few geologic archives that can preserve the atmosphere, such as amber (*9*) and fluid inclusions in minerals (*10*), although the robustness of these archives has been questioned (*11*).

Ice cores offer a unique opportunity to study past variations of *P*O_2_ because the ice directly traps the ancient atmosphere. This distinctive capability motivated early measurements of molecular oxygen in the trapped air (*12*, *13*). However, the gas-trapping process in ice leads to a small exclusion of O_2_ that has traditionally hindered the use of trapped O_2_ in ice cores to derive true atmospheric *P*O_2_, as the magnitude of this exclusion is comparable to that of the long-term *P*O_2_ change (see Supplementary Text for a more in-depth review). For example, Landais *et al.* (*14*) first observed in the trapped air of Dome C (EDC) ice cores a decline of the O_2_/N_2_ ratios (expressed as δO_2_/N_2_ hereafter; δ = *R*_sample_/*R*_standard_ – 1, where *R* is the elemental or isotopic ratio of interest and the standard is modern air) over the past 800 thousand years (ka), but the authors did not provide a conclusive explanation on the origin of this decline (i.e., a natural long-term δO_2_/N_2_ trend versus a storage artifact).

Additional data from three more deep polar ice cores (GISP2, Vostok, and Dome F) also exhibit a persistent decreasing trend in δO_2_/N_2_, the magnitude of which is −8.4 ± 0.2‰/million year (Ma) (1σ) (*8*). This trend has recently been corroborated by additional measurements on better preserved cores from EDC (*15*), indicating that the Late-Pleistocene δO_2_/N_2_ decline is not an artifact but a robust atmospheric signal (*8*). Because the atmospheric reservoir of N_2_ is expected to remain stable on time scales of hundreds of millions of years (*16*), the decline in ice core δO_2_/N_2_ is interpreted to reflect a decreasing *P*O_2_. Proposed drivers of this decline, as presented by Stolper *et al.* (*8*), include net oxidation of reduced carbon and sulfur associated with enhanced Pleistocene physical and chemical weathering (*17*) and/or the effects of decreased ocean temperature on organic carbon burial rates (*3*). The exposure of continental shelves during glacial sea-level lowstands has recently been identified as another potential candidate (*18*).

Here, we extend the ice-core record of atmospheric *P*O_2_ to ~1.5 Ma using “snapshots” of δO_2_/N_2_ in the air trapped in the glacial ice from the Allan Hills Blue Ice Area (BIA). The data include samples first reported by Yan *et al.* (*19*) and additional analyses conducted for the present study. We use the term snapshots to highlight the discontinuous nature of the blue ice samples and contrast that with the δO_2_/N_2_ time series obtained from continuous ice records (*8*, *15*). In Allan Hills BIA, ancient (up to 2.7 Ma) ice is transported toward the surface by a combination of glacial flow guided by the underlying bedrock topography and strong katabatic winds (fig. S1) (*19*, *20*). An absolute chronology of the ice is developed using the deficit of ^40^Ar compared to the modern atmosphere (termed ^40^Ar_atm_ and calculated as δ^40^Ar/^38^Ar – δ^38^Ar/^36^Ar) (*19*, *21*). δ^18^O of the trapped O_2_ provides additional stratigraphic links between two ice cores (fig. S2). Each individual δO_2_/N_2_ sample was assigned the age of the closest ^40^Ar_atm_ datum. Using this chronology, we divided samples into three distinct time bins: 1.5 Ma (± 0.1 Ma), 810 ka (± 100 ka), and 400 ka (± 70 ka). The numbers represent the average of ^40^Ar_atm_ ages weighted by their respective analytical uncertainty, bracketed by ±95% confidence intervals (CIs). The samples within each age bin do not necessarily have the exact same age. Rather, they are binned together because their age difference cannot be resolved by the existing ^40^Ar_atm_ dating method. This means that the true width of age distribution (data S1) is likely wider than the 95% CI shown in the brackets. This is to our advantage because integrating over longer time intervals filters out high-frequency variations in *P*O_2_ while preserving the long-term trend. Binning age groups this way allows the age uncertainty to be more rigorously defined and the effect of dating uncertainties on the long-term *P*O_2_ change to be quantified.

Reconstructing atmospheric *P*O_2_ from measurements of δO_2_/N_2_ of trapped air in ice cores requires corrections for fractionation during firnification and post-coring gas losses (*13*, *22*, *23*). In the study of Stolper *et al.* (*8*), corrections were achieved by first removing the insolation cycles in the δO_2_/N_2_ time series by linear regression and then by applying a constant gas-loss correction to δO_2_/N_2_ so each ice core δO_2_/N_2_ time series arrives at 0 when time is extrapolated to present. However, in the case of blue ice records from Allan Hills BIA, the insolation signals and gas losses cannot be directly accounted for because the age is not well resolved. In addition, the possibility of imperfect preservation of the δO_2_/N_2_ variability in a discontinuous record like blue ice implies that simple descriptive statistics (mean and variance) of the snapshots could be biased toward periods with high accumulation rates/better sample preservation (*19*). To reduce these confounding factors, we take advantage of the argon-to-nitrogen ratios (expressed as δAr/N_2_, again with modern air as the standard) in the trapped gases and use δAr/N_2_ as a proxy for fractionation modulated by insolation during firnification and fractionation due to post-coring gas loss.

**Fig. 1.**
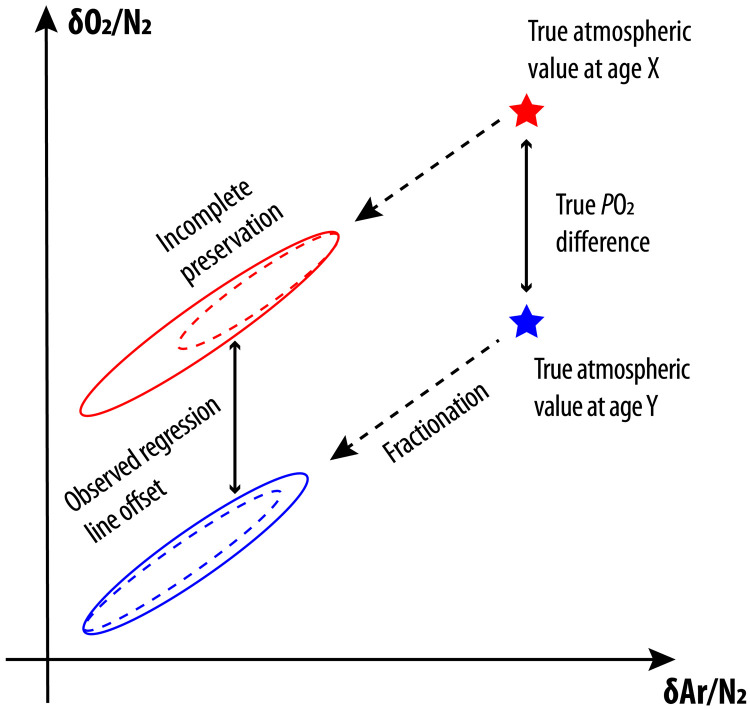
A schematic illustration of paired δO_2_/N_2_-δAr/N_2_ approach (not to scale). Suppose there are two populations of δO_2_/N_2_-δAr/N_2_ data with different ages (stars), different magnitude of gas fractionation (dashed arrows), and different degree of sample preservation (dashed circles). The true atmospheric δO_2_/N_2_ changes can be inferred from the offset in the observed δO_2_/N_2_-δAr/N_2_ data if (i) the slopes of δO_2_/N_2_ against δAr/N_2_ within the two populations are the same and (ii) there is no secular change in δAr/N_2_. The first assumption is supported by the empirical observation that the δO_2_/N_2_-δAr/N_2_ slopes exhibited in Allan Hills samples are statistically indistinguishable among three intervals. The second assumption is supported by a lack of long-term ice core δAr/N_2_ trend over the past 800 ka (*8*).

Ar and N_2_ are fractionated during bubble close-off and gas loss in a fashion similar to O_2_ and N_2_ but different in magnitude, leading to the covariation between δO_2_/N_2_ and δAr/N_2_ in ice cores (*13*, *22*, *23*). Part of this covariation arises from bubble close-off fractionation and is ultimately modulated by local insolation (*24*). As a result, correcting δO_2_/N_2_ using δAr/N_2_ measurements effectively removes the effect of local insolation on δO_2_/N_2_ during firnification. In the Supplementary Text, we show that post-coring gas loss can also be empirically accounted for by δAr/N_2_ corrections, although two other aspects of the δAr/N_2_ correction merit consideration here. First, due to the very small outgassing rate of ^40^Ar (0.066‰/Ma) (*21*) and the long residence time of N_2_ (*16*), atmospheric δAr/N_2_ variations will be far smaller than the changes in δO_2_/N_2_ inferred from ice cores and can thus be neglected. The effect of global ocean temperature on the solubility of Ar and N_2_ and atmospheric δAr/N_2_ values is also expected to be small (<0.2‰) and can also be neglected. Second, this method is expected to be robust to the partial preservation and recovery of the true range of δO_2_/N_2_ or δAr/N_2_ variability, as long as the slope of the δO_2_/N_2_-δAr/N_2_ data remains the same (Fig. 1). In this case, the offset in two populations of δO_2_/N_2_-δAr/N_2_ is interpreted to reflect changes in *P*O_2_.

## RESULTS

### Testing the paired δO_2_/N_2_-δAr/N_2_ approach using EDC and Vostok ice

**Fig. 2.**
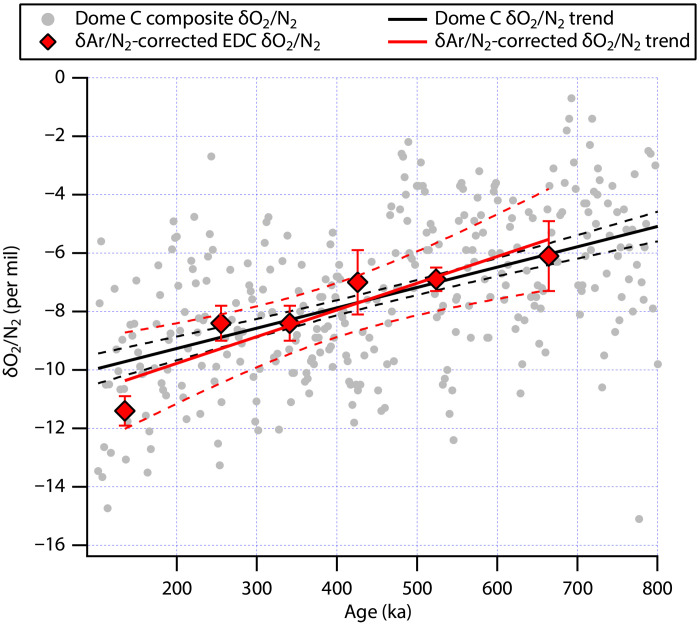
Testing the paired δO_2_/N_2_-δAr/N_2_ using the Dome C ice core gas records. A composite Dome C δO_2_/N_2_ (gray dots) compiled by Extier *et al.* (*15*) yields a temporal trend of −7.0 ± 0.6‰/Ma (1σ; shown as a solid line bracket by two dashed curves). The corrected δO_2_/N_2_ data (red squares with error bars representing the 95% CI) binned into six age groups (fig. S3) also yield a decreasing trend with a slope of −9.1 ± 2.7‰/Ma (1σ). Raw δO_2_/N_2_ and δAr/N_2_ data for correction are from Haeberli *et al.* (*25*).

To test the validity of this paired δO_2_/N_2_-δAr/N_2_ approach, we first used a recently published, gravitationally corrected dataset of δAr/N_2_ from EDC (*N* = 40) between 120 and 700 ka to correct the δO_2_/N_2_ data measured from the same ice (*25*). This dataset was obtained from ice stored at −50°C and is considered free from post-coring gas-loss fractionation. Six age bins were identified. In each age bin, δAr/N_2_ was normalized to −6.3‰, the average value of all δAr/N_2_ data in the record, to calculate δO_2_/N_2_ using the observed δO_2_/N_2_-δAr/N_2_ relationship in that bin (fig. S3). Except in the youngest (135 ka) group, the corrected δO_2_/N_2_ falls almost exactly on the trend line of δO_2_/N_2_ versus time [−7.0 ± 0.6 (1σ) ‰/Ma; Fig. 2] calculated from a high-resolution Dome C δO_2_/N_2_ record (*N* = 325) compiled by Extier *et al.* (*15*). Note that this high-resolution Dome C δO_2_/N_2_ record does not include the low-resolution δO_2_/N_2_ time series. The trend line observed in the low-resolution δO_2_/N_2_ record after δAr/N_2_ corrections is −9.1 ± 2.5‰/Ma (1σ). In spite of the relatively low temporal resolution of the data measured by Haeberli *et al.* (*25*), the δAr/N_2_ correction manages to reproduce the trend in δO_2_/N_2_, highlighting that this approach does not require the full or unbiased preservation of the true δO_2_/N_2_ and δAr/N_2_ variability in the ice.

Next, we applied the paired δO_2_/N_2_-δAr/N_2_ approach to the Vostok δO_2_/N_2_ record between 243.5 and 372.5 ka (*8*, *24*), which exhibits a temporal trend of −20.2 ± 3.3‰/Ma (1σ; fig. S4). We note that the trend in Vostok is steeper and with larger uncertainties than the rate inferred from the Dome C δO_2_/N_2_ record, possibly because of Vostok record’s relatively short time span (~130 ka) and hence a relatively large insolation signal here. We arbitrarily divided the Vostok gas data into two age groups: 243.5 to 300 ka (*N* = 27) and 300 to 372.5 ka (*N* = 29). When δAr/N_2_ is normalized to −4.8‰, the average value of all Vostok δAr/N_2_ data between 243.5 and 372.5 ka, the estimated δO_2_/N_2_ (± 2σ) is −8.9 ± 0.6‰ in the >300-ka group and −10.6 ± 0.5‰ in the <300-ka group. Using a time span of 64.5 ka (the difference between 271.75 and 336.25 ka), the inferred δO_2_/N_2_ rate of change is −26.0 ± 6.2‰/Ma (1σ). The δAr/N_2_ correction leads to about two times larger uncertainty for the final estimated δO_2_/N_2_ change rate, possibly due to the scatters in the δO_2_/N_2_ and δAr/N_2_ cross-plot. Another reason may be the short time span and the small magnitude of the change (hence the larger relative error).

To sum up, in both cases of EDC and Vostok ice, corrections by δAr/N_2_ are able to recover a rate of δO_2_/N_2_ change that is statistically indistinguishable from the rate of δO_2_/N_2_ changes regressed directly against time. This agreement justifies the application of paired δO_2_/N_2_-δAr/N_2_ approach in 1.5-Ma samples from the Allan Hills BIA.

### δO_2_/N_2_-δAr/N_2_ in Allan Hills ice samples

**Fig. 3.**
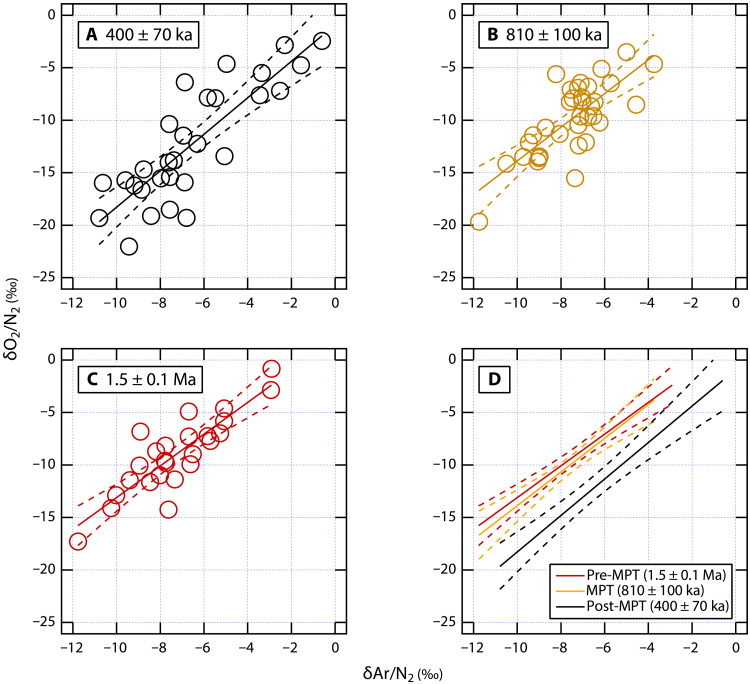
Cross-plot of δO_2_/N_2_ versus δAr/N_2_ measured in Allan Hills ice core samples, binned according to their age (error represents the 95% CI of the regression slopes). Solid lines in (**A**) to (**C**) represent the regression lines of δO_2_/N_2_ against δAr/N_2_, with the CI bracketed by the dashed lines. The regression lines of the three intervals are shown in (**D**) to demonstrate their offset.

**Fig. 4.**
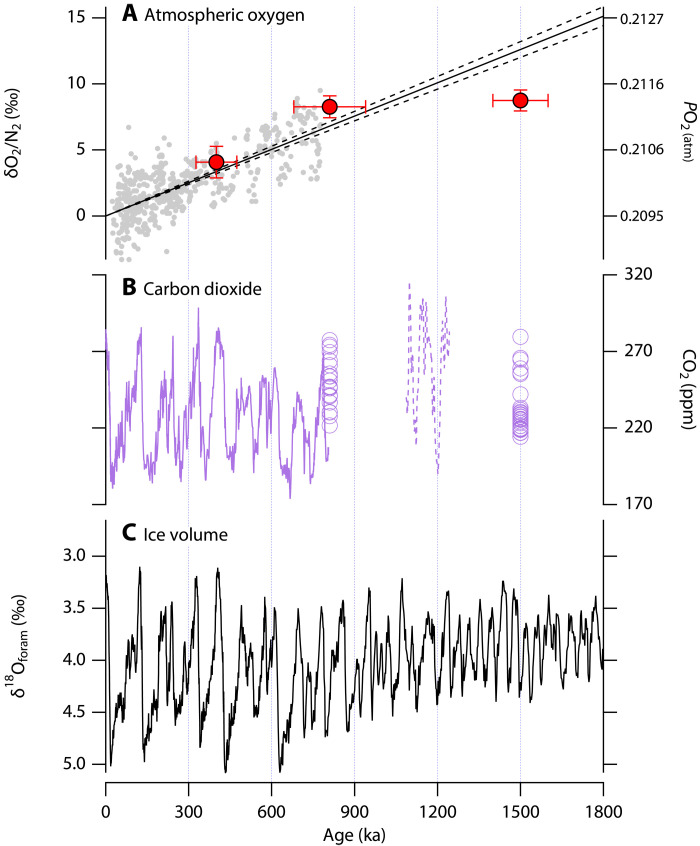
Pleistocene δO_2_/N_2_, *P*co_2_, and ice volume. (**A**) An ice core–based time series of atmospheric δO_2_/N_2_ composite (gray circles) (*8*) and discontinuous atmospheric δO_2_/N_2_ snapshots reconstructed from blue ice δO_2_/N_2_ (red circles, this work). Black line represents the extrapolated regression line of the continuous 800-ka δO_2_/N_2_ composite, with the 95% CIs bracketed by the two dashed lines. Vertical error bars associated with blue ice δO_2_/N_2_ are the 95% CIs of the corrected δO_2_/N_2_ when δAr/N_2_ is normalized to −7.1‰. Horizontal error bars represent the 95% CI of the age estimates. The blue ice δO_2_/N_2_ values were systematically increased by 17.4‰, so δO_2_/N_2_ reaches 0 when extrapolated from the 810- and 400-ka data to present, consistent with the treatment of the composite atmospheric δO_2_/N_2_ time series (*8*). (**B**) A continuous ice core CO_2_ record over 800 ka (solid purple line) (*64*–*67*), atmospheric CO_2_ reconstructions based on boron isotopes (dashed purple line) (*38*), and the Allan Hills blue ice CO_2_ data, grouped to the same age assignment as for δO_2_/N_2_ (purple circles) (*7*, *32*). (**C**) Stacked oxygen isotope composition of benthic foraminifera (LR04) (*30*).

δO_2_/N_2_ and δAr/N_2_ measured on Allan Hills ice samples and corrected for gravitational fractionation using the δ^15^N of N_2_ (*26*) are also linearly correlated (Fig. 3). In addition, we find that the slope of the regression line in the three age units is statistically indistinguishable (Table 1). This lack of difference suggests that there has been no fundamental change to the physical mechanisms that fractionate O_2_, Ar, and N_2_ over time in the Allan Hills ice, and provides an empirical justification for the use of δAr/N_2_ to correct Allan Hills δO_2_/N_2_ data for fractionation associated with bubble close-off and gas losses, and incomplete sampling of δO_2_/N_2_ variability associated with local insolation. Given the small difference in the slope of δO_2_/N_2_ against δAr/N_2_ across the three age units, we first normalize δO_2_/N_2_ values by correcting measured δAr/N_2_ value to −7.1‰, the average value of all δAr/N_2_ data (*N* = 88) reported in this study. Physically, this normalization means that the effect of insolation on δO_2_/N_2_ has been removed, and all the δO_2_/N_2_ data have the same extent of gas loss. This is different from normalizing δAr/N_2_ to 0, which would effectively eliminate all gas losses. Next, we applied a secondary gas loss correction by adding 17.4‰ to the estimated δO_2_/N_2_ to obtain atmospheric δO_2_/N_2_, conceptually similar to that by Stolper *et al.* (*8*). δO_2_/N_2_ should reach 0 when time is extrapolated to the present day (Fig. 4). This secondary correction does not change the slope inferred from the estimated δO_2_/N_2_ after δAr/N_2_ normalization. Extrapolating δAr/N_2_ to 0 (the theoretical true atmospheric value) for δO_2_/N_2_ correction does not change our results but is associated with larger uncertainties (see *y* intercepts in Table 1).

**Table 1.** Results of ordinary least squares linear regression of δO_2_/N_2_ against δAr/N_2_ measured in Allan Hills ice core samples. Errors are given as 95% CIs.

	**Post-MPT**	**MPT**	**Pre-MPT**
	**(400 ± 70 ka)**	**(810 ± 100 ka)**	**(1.5 ± 0.1 Ma)**
Slope	1.74 ± 0.44	1.60 ± 0.50	1.51 ± 0.38
*Y* intercept	−0.9 ± 3.1‰	2.2 ± 3.8‰	2.0 ± 2.9‰
Correlation coefficient *r*	0.84	0.76	0.86
Corrected δO_2_/ N_2_ (when δAr/ N2 = −7.1‰)	−13.3 ± 1.2‰	−9.2 ± 0.8‰	−8.7 ± 0.8‰

When normalized to the mean δAr/N_2_ value (−7.1‰), the corrected δO_2_/N_2_ is −8.7 ± 0.8‰ (95% CI; *N* = 25) in the 1.5 ± 0.1 Ma ice, −9.2 ± 0.8‰ (95% CI; *N* = 34) in the 810 ± 100 ka ice, and −13.3 ± 1.2‰ (95% CI; *N* = 29) in the 400 ± 70 ka ice (Table 1). The inferred rate of Late-Pleistocene δO_2_/N_2_ change, calculated by dividing the δO_2_/N_2_ difference in the 810 ± 100 ka and 400 ± 70 ka ice by their age difference, is −10.2 ± 2.5‰/Ma (1σ). The uncertainty is estimated by a Monte-Carlo simulation in which the division operations are repeated 10,000 times assuming a normal distribution for δO_2_/N_2_ and age according to their calculated 95% CIs. The uncertainty in our estimates is relatively large because our approach has fewer samples than the continuous δO_2_/N_2_ records obtained from deep ice cores and the ^40^Ar_atm_ ages of the blue ice introduce another layer of uncertainty. Nevertheless, the change in *P*O_2_ estimated from Allan Hills ice is statistically indistinguishable from the estimated change of −8.4 ± 0.2‰/Ma (1σ) diagnosed by Stolper *et al.* (*8*), supporting our paired δO_2_/N_2_-δAr/N_2_ approach.

In contrast, the calculated rate of *P*O_2_ change between the δO_2_/N_2_-δAr/N_2_ dataset recorded in the 1.5-Ma and 810-ka ice is −0.7 ± 0.9‰/Ma (1σ), statistically indistinguishable from zero. Because of the large size of the atmospheric O_2_ reservoir (3.8 × 10^19^ mol) and the relatively small O_2_ flux associated with geologic sources and sinks, the geologic residence time of atmospheric O_2_ is over 1 Ma (*27*), meaning that large *P*O_2_ fluctuations between 1.5 Ma and 810 ka unlikely result from geologic O_2_ sources or sinks. Some readers may identify photosynthesis and aerobic respiration in the biological cycling of oxygen as sources and sinks of O_2_, but the biosphere reservoir of organic carbon is two orders of magnitude smaller than the atmospheric O_2_ reservoir (fig. S5). For example, glacial-interglacial changes of terrestrial biosphere carbon reservoir size amount to ~3 to 7 × 10^16^ mol O_2_ (*28*), which would lead to a shift of the *P*O_2_ of ~1 to 2‰. To illustrate this point further, permafrost is a major source of unstable organic carbon reservoir and represents ~1 × 10^18^ g carbon in Earth’s near surface (0 to 3 m) (*29*). Oxidizing this entire surface reservoir would only lead to a 2‰ decline in atmospheric O_2_. We thus conclude that the initiation of the decline in atmospheric *P*O_2_ approximately coincided with the Mid-Pleistocene transition (MPT), a period between ~1250 and 700 ka where Earth’s climate underwent a fundamental shift characterized by the emergence of high-amplitude glacial-interglacial oscillations and an increase in the duration of glacial cycles from 40 to 100 ka (*30*–*32*).

## DISCUSSION

Assuming a geologic residence time of ~2 Ma (*27*), the decline in δO_2_/N_2_ over the last 800 ka is equivalent to a ~2% excess of O_2_ sinks over sources (*8*). These O_2_ sources and sinks include (i) the burial of reduced carbon, sulfur, and iron species into the sedimentary reservoir (geologic O_2_ source) and (ii) the oxidative weathering of the previously buried reduced carbon, sulfur, and iron species exhumed to Earth’s surface (geologic O_2_ sink). Because the total amount of O_2_ consumed over the last 800 ka (~2.4 × 10^17^ mol) is equivalent to ~1.2 times the combined size of the terrestrial and marine biosphere and soils (in O_2_ equivalents; fig. S5), changes in geologic sources and/or sinks must act to drive the long-term *P*O_2_ change.

Our new data, which almost doubles the length of the existing ice-core record of atmospheric O_2_ (*8*), indicates (i) an even slower rate of change of *P*O_2_ on a multimillion-year time scale and (ii) that the decline over the last 800 ka appears to have begun sometime around the MPT (Fig. 4). Before this, sources and sinks of O_2_ appear to have been in close balance. That the MPT marks the beginning of this small Late-Pleistocene imbalance in O_2_ sources and sinks is important for two reasons. First, the timing suggests a causal link between the MPT and a change in the global O_2_ balance favoring sinks over sources. This link, regardless of its ultimate cause(s), provides unique information about the processes that regulate atmospheric O_2_ on geologic time scales. Second, assuming that the global CO_2_ and O_2_ cycles are linked, *P*co_2_ (partial pressure of CO_2_) should begin to increase as soon as *P*O_2_ starts to decline. However, there is no apparent increase in atmospheric CO_2_ across the MPT (Fig. 4), which requires mechanisms to stabilize atmospheric *P*co_2_.

Across the MPT, the increase of global ice volume added up to ~60 m in sea-level equivalents, estimated from changes in benthic foraminiferal δ^18^O values (*30*, *33*, *34*). This volumetric increase appears to have been accommodated largely by an increase in ice sheet thickness, as geologic evidence indicates that the areal extent of Laurentide Ice Sheet has been similar since Early Pleistocene (*35*). In the meantime, mountain glaciers appear to have expanded considerably across the MPT in the Alps, Canadian Rockies, Tibetan Plateau, and Patagonia [(*36*) and references therein]. Deep-sea temperature changes across the MPT reconstructed from Mg/Ca ratios recorded in foraminiferal shells are ambiguous and inconclusive due to different regional trends (*33*, *37*). An alternate approach, which subtracts an independently reconstructed global ice-volume from the benthic δ^18^O stack, shows that there appears to be no substantial deep-sea cooling across the MPT (*34*). Interglacial CO_2_ levels remained somewhat stable before and after the MPT varying between 260 and 300 parts per million (ppm), but a decrease of glacial CO_2_ concentration minima by ~30 ppm (Fig. 4) was reconstructed from blue ice (*19*) and from boron isotopes in marine fossils (*38*).

Existing hypotheses on the Late-Pleistocene decline of O_2_ include (i) a decrease in the burial of reduced carbon and sulfur species linked to long-term ocean cooling and an increase in the solubility of O_2_ in the seawater [a hypothesis first put forward by Shackleton (*3*) to explain the Cenozoic history of O_2_], (ii) an increase in the oxidation of reduced carbon and sulfur species associated with lower sea-level during glacial maxima and the exposure of recently deposited marine sediments (*18*), and/or (iii) an increase in the oxidation of reduced carbon and sulfur species relative to enhanced organic carbon and pyrite burial due to higher erosion rates (*8*).

Ocean cooling over the Pleistocene [hypothesis (i)] is possible, although it is not firmly established from global deep-sea temperature records (*33*, *34*) or marginal marine environments where most of the reduced carbon and sulfur is buried. In contrast, increased oxidation of reduced carbon and sulfur associated with increased exposure of the continental shelf during the sea-level lowstands of the Late Pleistocene [hypothesis (ii)] can explain ~70% of the Late-Pleistocene *P*O_2_ decline (*18*). However, this estimate is likely an upper limit, because the calculation is based on pyrite exposed by a ~90-m sea-level difference in the Late-Pleistocene glacial cycles. If we apply the same mechanism given the glacial-interglacial sea-level difference of ~60 m before the MPT, either *P*O_2_ declined at a slower rate of −4‰ to −5‰/Ma in the Early Pleistocene (not consistent with observation) or only the deeper pyrite newly exposed by the additional 30-m sea-level drop after the MPT acted to break the balance between oxygen sources and sinks. In the latter case, the expected effect of continental shelf pyrite exposure and oxidation will only be able to account for ~21% of the observed Late-Pleistocene atmospheric O_2_ decline.

Increased oxidation of reduced carbon and sulfur associated with increased Late-Pleistocene continental glaciation and erosion [hypothesis (iii)] is therefore more likely to have contributed to the post-MPT δO_2_/N_2_ decline. For example, a link between more extensive post-MPT mountain glaciation and an increase in erosion rates is observed at St. Elias Mountains, Alaska (*39*). In addition, glacial weathering elevates pyrite oxidation that acts as a net O_2_ sink (*40*) and increases the oxidation rates of fossil organic carbon in rocks by a factor of 2 to 3 in glaciated mountainous regions due to enhanced frost cracking, abrasion, oxygen availability in deeper soil horizons, and microbial activity (*41*). All these glaciation-facilitated processes could enhance O_2_ sinks, thereby leading to a *P*O_2_ drawdown since the MPT with the presence of larger ice sheets. We note that increasing erosion rates also promote organic and pyrite burial, and the net effect on *P*O_2_ remains not fully resolved (*42*). In any case, both hypotheses (ii) and (iii) suggest that the MPT could not only reshape the glacial-interglacial cycles but also profoundly affect the biogeochemical cycles of carbon and oxygen.

Last, a decline in *P*O_2_ due to changes in organic carbon burial and oxidation would inject CO_2_ to the coupled atmosphere-ocean system. Even if that decline is entirely due to the enhanced pyrite oxidation, atmospheric CO_2_ would still increase—albeit with a smaller magnitude due to interactions with the inorganic carbon cycle. When coupled with calcium carbonate dissolution, pyrite oxidation has a stoichiometric 8:15 ratio of CO_2_ produced to O_2_ consumed (*18*). This is approximately half the yield of CO_2_ during oxidation of organic carbon, assuming the composition of the organic carbon is CH_2_O (i.e., 1 mol of CO_2_ is produced when 1 mol of O_2_ is consumed). Only when all the H^+^ produced during pyrite oxidation is consumed by silicate dissolution can *P*co_2_ be truly decoupled from the oxygen cycle (*40*).

Records of atmospheric CO_2_ from both ice cores (*19*) and boron isotopes in foraminifera (*38*) indicate that average CO_2_ levels either did not substantially change or actually declined across the MPT (Fig. 4). As noted by Stolper *et al.* (*8*), this observation can be explained by the *P*co_2_-dependent silicate weathering feedback with a 200- to 500-ka response time. However, we note that with even the fastest response times *P*co_2_ must still increase in order for the feedback to establish a balance between sources and sinks [figure 3 in the study of Stolper *et al.* (*8*)]. This instantaneous increase in *P*co_2_ was ignored by Stolper *et al.* (*8*) because it only occurs following the initiation of a decline in *P*O_2_ and the timing of that initiation was not resolved in the 800-ka *P*O_2_ record. Our ability to identify the MPT as the initiation of a decline in *P*O_2_ means that this initial increase in CO_2_ can no longer be ignored. Additional responses and/or enhanced feedbacks within the global carbon cycle are required to explain why CO_2_ remained constant or declined across the MPT. Although speculative, two promising candidates are (i) enhanced CO_2_ storage in the deep Atlantic Ocean (*43*) enabled by seafloor calcium carbonate dissolution (*44*) and linked to ocean circulation changes (*45*) and (ii) enhanced chemical weathering resulting from the transition from regolith to fresh, unweathered crystalline bedrock beneath the Laurentide Ice Sheet that became first exposed around the MPT (*31*).

Oxygen-to-nitrogen ratios in the air bubbles trapped in blue ice from Allan Hills, East Antarctica provide a record of atmospheric O_2_ extending to the Early Pleistocene after correction by argon-to-nitrogen ratios preserved in the same sample. This correction approach is tested on the Vostok and Dome C δO_2_/N_2_ records and successfully reproduces their long-term trends. A decline in *P*O_2_ inferred from 810- and 400-ka Allan Hills ice samples agrees with *P*O_2_ reconstructions based on continuous ice records (*8*, *15*), further supporting the δAr/N_2_ correction method. There is no statistically significant δO_2_/N_2_ difference between samples at 1.5 Ma and 810 ka, suggesting a stable mean O_2_ level and a balanced O_2_ sources and sinks before the MPT. The coincidence of *P*O_2_ decline with the MPT hints at the role of glaciation that enhances erosion rates as well as promotes the oxidative weathering of sedimentary organic carbon and reduced sulfur species. The lack of transient *P*co_2_ increase in response to the declining *P*O_2_ requires responses in the carbon cycle to stabilize atmospheric CO_2_, possibly first via a fast increase of deep ocean carbon storage as a result of ocean circulation changes (*43*, *45*) and in the long term by an enhanced silicate weathering due to the exposure of pre-Cambrian crystalline bedrock underneath the Laurentide Ice Sheet starting around the MPT (*31*).

## MATERIALS AND METHODS

### Glaciological settings

Allan Hills (76.73°S, 159.36°E) is located ~100 km to the northwest of the McMurdo Dry Valleys in Victoria Land, East Antarctica. Sampling sites are located to the west of the Allan Hills nunatak in the Main Ice Field (MIF; fig. S1). Samples reported in this study come from two boreholes in the MIF with close proximity: ALHIC1502 (76.73286°S, 159.35507°E) and ALHIC1503 (76.73243°S, 159.35620°E). Local glaciological conditions in the MIF are documented elsewhere (*46*–*48*), and Dadic *et al.* (*49*) provide a detailed survey of meteorological conditions and surface snow properties. In brief, the Allan Hills area is characterized by constant katabatic wind, sweeping away shallow snow patches and ablating the ice at the surface (*48*). The presence of blue ice near Allan Hills is further facilitated by a nunatak obstructing the upcoming glacial flow and a steep slope on the lee side of a mountain buried under the ice (*20*, *50*). The steep bedrock slope and low basal temperature inhibit glacial flow, thereby promoting the preservation of exceptionally old ice. Although modern-day glaciological survey reveals a local accumulation regime (*51*), the source region of the ice buried at depth in the Allan Hills BIAs remains unclear.

While the glaciological conditions favor the preservation of old ice here, they also pose several challenges to using blue ice as paleoclimate archives. First, the unique glaciological dynamics at the BIAs make it difficult to constrain the chronology of the ice, as ice movement has likely disturbed the otherwise continuous ice stratigraphy. If the age of the blue ice samples is within 800 ka, they could be synchronized to the deep ice cores via various age markers (*52*). However, if the age exceeds 800 ka, which is the case of Allan Hills blue ice, there is no existing record to synchronize to. An absolute dating method that does not require prior glaciological information is needed. In the section below, we describe the time scale development using gravitationally corrected ^40^Ar/^38^Ar ratio (^40^Ar_atm_).

### Time scale development

We established the blue ice chronology by measuring the gravitationally corrected ^40^Ar/^38^Ar ratios (^40^Ar_atm_, calculated as δ^40^Ar/^38^Ar – δ^38^Ar/^36^Ar) in the trapped gases (*21*). Individual ^40^Ar_atm_ datum and the corresponding uncertainty is included in data S1. This method takes advantage of the fact that atmospheric ^40^Ar burden slowly increases over time because of the radioactive decay of ^40^K in the solid Earth, while ^38^Ar and ^36^Ar are primordial and their abundance is stable over geologic history. The rate of ^40^Ar_atm_ change is +0.066‰/Ma over the past 800 ka (*21*), and we caution that the outgassing rate could have changed before the MPT and therefore changed our extrapolation results. If weathering rates have increased since the MPT due to more extensive glaciation, the outgassing rate of ^40^Ar is expected to be lower in the Early Pleistocene than in the Late Pleistocene. In this scenario, the >800-ka samples discovered at the Allan Hills BIA would have an even older age. This age underestimation means that the 1.5-Ma data point in Fig. 4 would need to be moved to the right, but the conclusions of this study would remain the same because δO_2_/N_2_ is essentially constant between the 810-ka and 1.5-Ma age bins.

In addition, because the depths of δO_2_/N_2_ samples are different from those of ^40^Ar_atm_ samples, in most cases, the age of δO_2_/N_2_ samples was assigned to their closest ^40^Ar_atm_ datum reported by Higgins *et al.* (*53*) and Yan *et al.* (*19*). Note that the analytical uncertainties associated with the ^40^Ar_atm_ measurements (at least 10% of the sample age) preclude an age assignment precise enough to establish a time series. To maximize the utility of the ^40^Ar_atm_ data, we classify samples into different age groups and calculate the average age ($\hat{\mu }$) on the basis of individual ^40^Ar_atm_ measurements (*x_i_*), weighted by their corresponding uncertainty (σ*_i_*)

$$\hat{\mu }=\frac{\sum x_{i}/\sigma_{i}^{2}}{\sum 1/\sigma_{i}^{2}}$$ (1)

The error associated with the weighted mean is given by

$$\sigma^{2}(\hat{\mu })=\frac{1}{\sum 1/\sigma_{i}^{2}}$$ (2)

Underlying this error estimation is the implicit assumption that all samples within the age bin have the exact same age and the precision of age estimates is purely limited by the analytical precision of ^40^Ar_atm_. We acknowledge that there is no independent evidence to support this assumption. That being said, the purpose of such age binning is to provide a quantifiable age uncertainty that can then be taken into consideration in calculating long-term *P*O_2_ trend. Basing these calculations on the difference between the average age of two or more intervals has proven valid in EDC (fig. S3) and Vostok (fig. S4). In any case, uncertainties associated with age will affect the estimated rate of *P*O_2_ decline in the Late-Pleistocene, but will not change the conclusion of a stable atmospheric O_2_ level before the MPT.

Three age groups were identified and listed in Table 1: pre-MPT, MPT, and post-MPT, in accordance with the definition by Yan *et al.* (*19*). The post-MPT group has an average ^40^Ar_atm_ of 400 ± 70 ka (95% CI) from 23 ^40^Ar_atm_ measurements made on 20 depths in ALHIC1502 and ALHIC1503 cores. Similarly, the mean age of the MPT group is calculated to be 810 ± 100 ka (95% CI), constrained by nine ^40^Ar_atm_ data points from six depths. The pre-MPT age group has three sub-age units: 1.5, 2.0, and 2.7 Ma. In this study, we only include samples in the 1.5-Ma subgroup, the reason of which is discussed in the “Data quality and rejection criteria” section. This age (1.5 ± 0.1 Ma; 2σ) is estimated by four unreplicated ^40^Ar_atm_.

In the upper 170 m of ALHIC1502, there are only four ^40^Ar_atm_ data that provide age constraints. The transition from post-MPT to MPT age unit in ALHIC1502 is located between 143.19 and 172 m, the last post-MPT and the first MPT ^40^Ar_atm_-based age estimates, respectively. This data gap requires alternative estimates to identify the transition from post-MPT to MPT ice samples. By comparison, the chronology of ALHIC1503 is better constrained by 33 ^40^Ar_atm_ analyses from 27 depths. We assume that ALHIC1502 and ALHIC1503 have an overall similar age-depth structure given their close proximity, and the variations in δ^18^O_atm_ can be used as a marker to identify the depth at which MPT ice first appears in ALHIC1502. In ALHIC1503, the transition from post-MPT to MPT age unit occurs along with a rapid δ^18^O_atm_ decline from 1.361 to 0.488‰ (*53*). In ALHIC1502, this transition occurs between 146.40 and 147.99 m (fig. S2 and data S2). We therefore choose the midpoint in between (147.20 m) as the arbitrary divide between post-MPT and MPT ice. Note that there are no δO_2_/N_2_ data points between 146.40 and 147.99 m in ALHIC1502, so the conclusion of this paper is not affected by where this divide is exactly chosen.

Another interesting feature of the depth profile of ^40^Ar_atm_ in ALHIC1502 is the rather young (~880 ka) age in the deepest measured sample with a depth of 190 m. In contrast, the deepest ^40^A_ratm_ measurement in ALHIC1503 yields an age of 2.7 ± 0.3 (1σ) Ma. There are two possible explanations: (i) The ice dates back to the MPT and is buried under the pre-MPT ice due to stratigraphic disturbance or (ii) the age is underestimated because of input of radiogenic ^40^Ar from the bedrock. We have no way of identifying the true cause(s), but either way, it would not affect the conclusion of this paper because the δO_2_/N_2_ data were rejected on other bases (see the “Data quality and rejection criteria” section).

### Analytical methods for δO_2_/N_2_ and δAr/N_2_

The analytical procedures of δO_2_/N_2_, δAr/N_2_, and δ^15^N of N_2_ data reported in this study are modified from Dreyfus *et al.* (*54*) and described in detail by Yan *et al.* (*19*). A brief summary is outlined below. A total of ~20-g ice was cut from the core, with the outer 2 to 3 mm removed and subsequently melted under vacuum. To achieve that vacuum, the ambient air inside the glass container that carried the ice was pumped away for 3 min by a turbomolecular pump with the container placed in a dry ice–isopropanol bath. The ~200-ml glass container has a Louwers-Hapert valve at the top and an Ace-Thred connection sealed by a Viton O-Ring. After ice melted, the glass vial was loaded onto an electromechanical rotator to let the air and meltwater equilibrate (*55*). After 4 hours, the glass vial was taken down from the rotator, connected to a Büchner flask upside down, and drained by a rotatory pump. Then, the vial was flipped upside down and placed in a −30°C bath to let residual water refreeze. Gases in the headspace were cryogenically collected at 4 K in a stainless steel dip tube submerged in liquid helium. During the gas collection, H_2_O and CO_2_ were removed by two sequential gas traps, one kept at −100°C and the second placed in liquid nitrogen bath.

The dip tube was allowed to warm up at room temperature for at least an hour. Later, it was attached to an isotope-ratio mass spectrometer (Thermo Delta Plus XP) for elemental and isotopic analysis for ~30 min. The configuration of the mass spectrometer permits simultaneous measurement of mass 28 (^14^N^14^N) to mass 44 (^12^C^16^O_2_), thereby yielding raw ^15^N/^14^N, O_2_/N_2_, Ar/N_2_, and ^18^O/^16^O ratios at the same time. Analysis of each single sample was made up of 24 individual cycles, each with a 16-s integration time. The raw δ^15^N, δ^18^O, δO_2_/N_2_, and δAr/N_2_ ratios were corrected for pressure imbalance based on the ion currents on the sample and reference sides (*12*). Pressure-corrected δ^15^N and δ^18^O were further corrected for the elemental composition of the O_2_-N_2_ mixture (termed “chemical slope correction”) (*12*). After pressure imbalance and chemical slope corrections, pressure and chemically corrected δ^15^N was used to correct for gravitational fractionation in δO_2_/N_2_, δAr/N_2_, and δ^18^O of O_2_ (termed δ^18^O_atm_). Those gravitationally corrected data, presented in data S2, are used for interpreting atmospheric O_2_ concentrations.

The final pooled SDs of all Allas Hills δO_2_/N_2_, δAr/N_2_, and δ^18^O_atm_ [excluding those reported by Higgins *et al.* (*53*) because of their procedural differences] are ±3.372, ±2.081, and 0.027‰, respectively. All δ values have been standardized to the modern atmosphere. We note that the analytical uncertainties of Allan Hills δO_2_/N_2_ reported here, by Yan *et al.* (*19*), and by Higgins *et al.* (*53*) are worse than the reproducibility of the same properties achieved in other deep ice cores, which is well noted by Stolper *et al.* (*8*) and led to the exclusion of 1 Ma Allan Hills blue ice δO_2_/N_2_ data in that study. For example, the reproducibility of δO_2_/N_2_ measured on EDC and Dome Fuji ice samples is 0.37‰ (*15*) and 0.2‰ (*56*), respectively. This difference in reproducibility likely arises from the fact that gases are trapped in bubbles in the Allan Hills ice, whereas the EDC and Dome F δO_2_/N_2_ data were measured on ice that exclusively contains clathrate. Similar conclusions are drawn from Vostok samples, where gases contained in bubbles exhibit more scatter in measured δO_2_/N_2_ than gases from bubble-free, all-clathrate ice (*57*). It may be the case that bubbles are more susceptible to gas loss and fractionation as compared to clathrates. At any rate, the difference of bubbles versus clathrates means that Allan Hills data cannot be directly compared against other deep ice core data. Internal comparison between Allan Hills samples (all with bubbles only) of different ages is still justified. We acknowledge that the estimated δO_2_/N_2_-δAr/N_2_ slope and intercept derived from ice with bubbles must have larger uncertainty than does the slope and intercept derived from ice with clathrates only, but we expect the estimate to be unbiased, which is important for estimating long-term *P*O_2_ changes.

Here, we quantitatively investigate how much the observed δO_2_/N_2_ reproducibility is limited by the analytical precision associated with mass spectrometer analyses, using the 25 samples (all with replication) dating back to 1.5 Ma as an example. With a pooled SD of ±3.372‰ (1σ), 25 unique samples each with replication should yield a 95% CI of ±0.95‰, if all the disagreements between the δO_2_/N_2_ replicate values arise from the mass spectrometer measurements. The calculation is as follows: The observed pooled SD (3.372‰) is first divided by the square root of 2 to take replicate analyses into account, then divided by the square root of 25, the number of unique samples, and finally multiplied by 2 to yield the 95% CI. However, the 95% CI of the δO_2_/N_2_-δAr/N_2_ regression line is ±0.79‰ at δAr/N_2_ = −7.1‰, less uncertain than what pure analytical uncertainties alone would dictate. This observation means that correction by δAr/N_2_ reduces the uncertainties in the δO_2_/N_2_ dataset. Thus, the large analytical uncertainty of δO_2_/N_2_ measured on ice with bubbles must partly be sourced from processes that fractionate both δO_2_/N_2_ and δAr/N_2_, such as the different extent of gas losses experienced by the replicates during handling. The CI of the regression line and the estimated δO_2_/N_2_ can certainly benefit from improved analytical precision in the future.

To investigate how large analytical uncertainties could potentially affect the estimate of δO_2_/N_2_, we used a “York Fit” algorithm that takes into account the inherent uncertainty in both the independent (δAr/N_2_) and dependent (δO_2_/N_2_) variables in a generalized linear model (MATLAB code available at www.mathworks.com/matlabcentral/fileexchange/26586-linear-regression-with-errors-in-x-and-y) (*58*). We chose the pooled SD to represent the uncertainty associated with δAr/N_2_ and δO_2_/N_2_ data. The results are shown in fig. S6. In all three intervals (post-MPT, MPT, and pre-MPT), the York Fit yields a steeper slope, but none is significantly different from the slope yielded by the ordinary least squares (OLS) regression method used in our analysis. For the post-MPT samples, the York Fit slope is 2.16 ± 0.84 (2σ; same as below) with a *y* intercept of 1.9 ± 5.9‰, compared to the slope of 1.74 ± 0.44 and the *y* intercept of −0.9 ± 3.1‰ yielded by the OLS regression. For the MPT samples, the York Fit slope and *y* intercept are 2.31 ± 1.40 and 7.4 ± 10.6‰, respectively, while the OLS regression slope and *y* intercept are 1.60 ± 0.50 and 2.2 ± 3.8‰, respectively. The MPT samples have the largest departure of York Fit from the OLS fit. Last, for the pre-MPT samples, a slope of 1.77 ± 1.00 and a *y* intercept of 3.9 ± 7.5‰ are obtained from the York Fit, whereas the OLS slope and *y* intercept are 1.51 ± 0.38 and 2.0 ± 2.9‰, respectively.

Despite the changes to the slope when analytical uncertainties are taken into account, the estimated δO_2_/N_2_ when δAr/N_2_ is normalized to −7.1‰ does not change much (table S1), visually represented by the two types of regression lines intersecting near δAr/N_2_ = −7.1‰ (fig. S6). The corrected δO_2_/N_2_ (± 2σ) from York Fit is −13.47 ± 1.68, −8.96 ± 1.04, and −8.69 ± 1.06 for post-MPT, MPT, and pre-MPT samples, respectively. These results are very close to the OLS estimates made in the main text (table S1), because the value of −7.1‰ (the mean of all δAr/N_2_ values) is very close to the mean values of δAr/N_2_ in each age group. Visually, the York Fit tilts the slope of OLS regression around the arithmetic mean of the independent variables (δAr/N_2_). The estimated δO_2_/N_2_ near the arithmetic mean of δAr/N_2_ is therefore not substantially changed, but extrapolating δO_2_/N_2_ to δAr/N_2_ = 0 would lead to large differences (table S1). For these reasons, we still chose to regress δO_2_/N_2_ to δAr/N_2_ = −7.1‰ using the OLS method while acknowledging its inadequacy in addressing uncertainties in the variables. This facilitates the comparison of our regression results with the slopes of earlier work, all of which were done using the OLS method without factoring in the uncertainties in δO_2_/N_2_ or δAr/N_2_.

### Data quality and rejection criteria

There are three batches of δO_2_/N_2_ and δAr/N_2_ dataset measured on ice collected from the Allan Hills BIA. The first batch was measured in 2013–2014 on samples drilled in 2010–2011 Antarctic field season. This batch of data (“batch 1”) was first reported by Higgins *et al.* (*53*) with the method described by Dreyfus *et al.* (*54*). The second batch was measured in 2017 on samples collected in 2015–2016 Antarctic field season, with an updated gas extraction method described in this study. This batch of data (“batch 2”) has been reported by Yan *et al.* (*19*). The third and final batch (“batch 3”) was measured in 2018 and 2019, again with the updated method and on samples drilled in 2015–2016. This study presents the batch 3 data for the first time.

The key procedural distinction of batch 2 and 3 samples from batch 1 samples is that the pumping time has been greatly reduced. In batch 1 protocols, ice samples were pumped for an indefinite and variable amount of time until a vacuum level of 0.7 mtorr was reached. Each sample may therefore have experienced a varying degree of gas losses associated with storage and handling. The reduced pumping time applied to batch 2 and 3 data was intended to minimize gas losses due to pumping under vacuum and, if the losses are inevitable, to at least make the fractionation due to gas losses during pumping as consistent as possible. Note that this reduced pumping time does not prevent gas losses during storage and through fractures (if present).

In addition to analytical procedures, the conditions under which batch 1 to 3 samples are stored are also different. Specifically, batch 1 data were obtained from ice samples stored at a −25°C freezer for more than 2 years, and certain samples have visible internal fractures. In contrast, the δO_2_/N_2_ and δAr/N_2_ data reported in the present study come exclusively from ice cores drilled in the 2015–2016 field season. They have been stored at −36°C in the National Ice Core Facility in Denver, CO until retrieval for cutting and processing shortly before laboratory analyses and have no surface cracks or fractures. These storage and procedural differences may help explain the larger scatter observed in the δO_2_/N_2_-δAr/N_2_ data reported by Higgins *et al.* (*53*) made on samples dating back to the MPT (fig. S7). Batch 1 data were therefore excluded from our analysis (though we still present them in data S2, marked as batch 1). Batch 2 and 3 data are considered equivalent in terms of the data quality.

One post-MPT sample was rejected from batches 2 and 3 because of its very negative δAr/N_2_ value (−15.8‰). The δO_2_/N_2_ associated with this sample is −26.3‰, substantially (>5‰) more negative than typical ice core values. These anomalous negative ratios may arise from considerable gas loss experienced by this particular sample that greatly depletes Ar and O_2_ relative to N_2_. Six additional post-MPT samples were rejected because they are bracketed by pre-MPT age bins and might represent a different time slice than the shallow post-MPT samples. We still list these samples in data S2 but mark them as “anomalous gas loss” and “stratigraphy,” respectively. Including these data points in the regression analysis does not change the conclusion of this paper.

In addition to gas losses, aerobic respiration and abiotic oxidation could affect the molecular oxygen concentrations in the trapped air. Even if O_2_ consumption does not occur in situ inside the ice being measured, the large O_2_ gradient could potentially drive a diffusive flux and modify the ice core δO_2_/N_2_. Many 2.0-Ma and all 2.7-Ma samples come from the sections are affected by respiration, evidenced by the depleted δ^13^C values and anomalously elevated CO_2_ levels (*19*). Thirteen δO_2_/N_2_ samples in batches 2 and 3, including one that had already been excluded, were rejected on this basis (labeled “respiration” in data S2). There are only four 2.0-Ma samples that are considered “pristine” (i.e., not affected by respiration). These four pristine δO_2_/N_2_-δAr/N_2_ data points fall within the envelop defined by the rest of the Allan Hills blue ice δO_2_/N_2_-δAr/N_2_ data, but they were excluded from inferring Early-Pleistocene *P*O_2_ levels because of the small number of samples (annotated “2 Ma samples” in data S2). O_2_ consumption by nonmicrobial redox reactions such as Fe(II) oxidation has been observed in the nearby (~500 km) Talos Dome ice core (*59*). In the case of Allan Hills samples, this process is expected to generate negligible impact on δO_2_/N_2_, because of the low dust loading in the vicinity of Allan Hills [<700 parts per billion (ppb) during glacial and <15 ppb during interglacial periods] (*60*) and the low concentration of iron in the aeolian dust (~3% by mass).

In the case of very old ice here, an additional concern is the molecular diffusion through the ice that is capable of smoothing the signals in the gas record even after gases have become fully locked-in (*61*). This concern was partly motivated by the reduced variability observed in the portion of ALHIC1502 gas records below 150 m (fig. S2) and has been addressed in greater length by Yan *et al.* (*19*). We note that the variability reduction of δ^18^O_atm_ bears some resemblance to the reduced variability of δ^18^O_atm_ (among other properties) in the basal EDC ice below 3200 m (*62*). In the basal EDC record, the homogenization of dissolved impurities profile was hypothesized to arise from “accelerated migration re-crystallization, which results in spatial chemical sorting of the impurities” (*62*) under ice temperature close to the pressure melting point. The lack of δ^18^O_atm_ variability between 3200 and 3260 m in EDC was not entirely understood, however, because if mechanical modification (stretching and annual layer thickening) was the reason, the 60-m layer would need to span an unlikely ~10 ka in time (*62*). An alternative possibility to stretching is diffusive smoothing, which has been shown to be capable of removing the submillennial-scale δD_ice_ variability (*63*) and would explain the lack of variability while preserving the mean values. It could be the case that the basal EDC δD_ice_ and δ^18^O_atm_ have been subject to extensive diffusive smoothing and therefore lost its variability on multimeter length scale. If so, the extent of water isotope diffusion should be greater than that of gas diffusion (*61*). In the case of Allan Hills, calculations do not support the presence of molecular diffusion on the basis that δD of the ice shows considerable variability (*19*).

To summarize, all data measured on batch 1 samples and 23 data points from batches 2 and 3 were excluded. We retain a total number of 88 samples (each with at least one replicate) for calculations performed in this study.

## Supplementary Materials

### This PDF file includes:

Supplementary Text Figs. S1 to S8 Table S1 Legends for datasets S1 and S2 References

### Other Supplementary Material for this manuscript includes the following:

Datasets S1 and S2
