# Supplementary material for: Ice core evidence for atmospheric oxygen decline since the Mid-Pleistocene transition
Source: Sci Adv. 2021 Dec 15;7(51):eabj9341. doi: 10.1126/sciadv.abj9341 (PMC8673763; doi:10.1126/sciadv.abj9341)
Supplement: Supplementary file 1 — Supplementary Text Figs. S1 to S8 Table S1 Legends for datasets S1 and S2 References [file sciadv.abj9341_sm.pdf]

Science Advances
AAAS

Supplementary Materials for

Ice core evidence for atmospheric oxygen decline since the Mid-Pleistocene transition

Yuzhen Yan*, Edward J. Brook, Andrei V. Kurbatov, Jeffrey P. Severinghaus, John A. Higgins

*Corresponding author. Email: yuzhen.yan@rice.edu

Published 15 December 2021, *Sci. Adv.* **7**, eabj9341 (2021)
DOI: 10.1126/sciadv.abj9341

The PDF file includes:

Supplementary Text
Figs. S1 to S8
Table S1
Legends for datasets S1 and S2
References

Other Supplementary Material for this manuscript includes the following:

Datasets S1 and S2

Supplementary Text

Reconstructing past atmospheric O_2 concentration variations was one of the major inspirations of some pioneering efforts to measure the elemental composition of the trapped gases in ice cores in the late 1980s. However, Ar/N_2 and O_2/N_2 ratios in ice cores were found to be 5-10‰ lower than the true atmosphere values (*12*). It was soon realized that physical processes have altered the composition of the trapped gases from its true atmospheric value (*22*, *26*, *68*, *69*). Until lately, these processes have precluded the derivation of atmospheric oxygen concentrations from ice cores. We briefly describe the process of gas trapping and the origin of the covariation between $\delta O_2/N_2$ and $\delta Ar/N_2$ below, which is the theoretical foundation of correcting $\delta O_2/N_2$ by $\delta Ar/N_2$.

Gases are "captured" during the densification of the snow with the growth of ice crystals. At the very onset, fresh snow on the surface of a glacier forms a low-density, porous, convective layer in which free air motion does not cause fractionation. Older snow layers are progressively buried and compacted by subsequent, younger depositions. The compaction causes the porosity to decline and the density to rise with depth, forming what is known as a "firn" layer. Inside the firn, the movement of the gas molecules transitions from advection in the uppermost part of the firn to molecular diffusion below. Within the diffusive zone, heavy species are enriched at greater depth of the gravity column (*26*) in the absence of temperature gradients that thermally drive heavy isotopes and molecules towards the cold end (*68*). At depth, the ice eventually becomes impermeable to gas diffusion due to the ever-increasing overloading pressure. In terms of gas trapping, the critical step occurs when the overburdening pressure drives the firn density to cross a threshold value of ~800 kg/m^3 (*70*). On the microscopic level, this step is characterized by the ice lattice shrinking to a threshold size where molecular motion is impeded. This process is termed "bubble close-off", as the closure of air bubbles "locks" the gases inside. Larger molecules such as N_2 will first become

locked while smaller molecules like Ar, O_2, Ne, and He can still move within the firn. However, even their motion would eventually be inhibited when ice lattice size is further reduced. A critical size of the ice lattice at which close-off fractionation of O_2/N_2 and Ar/N_2 begins is 0.36 nm, concluded by multiple studies (*69*, *71*, *72*). The magnitude of this bubble close-off fractionation appears to be strongly size-dependent, evidenced by the observation that Ne is fractionated 34 ± 2 times than O_2 in South Pole firn air (*69*).

It is worth noting that before the air motions inside the firn were constrained by detailed observations, ice core $\delta O_2/N_2$ was empirically found to have a high coherency with local summertime insolation (*24*). The subtle influence on ice grain properties by insolation at the time of snow deposition is thought to modulate the magnitude of bubble close-off fractionation (*73*). Since then, this empirical relationship between $\delta O_2/N_2$ and local summer insolation makes ice core $\delta O_2/N_2$ a useful tool of orbital tuning to constrain ice core chronologies (*24*, *57*). $\delta Ar/N_2$ also covaries with insolation and have shared variance with $\delta O_2/N_2$ [Fig. S1 in Stolper *et al* (*8*)]. Indeed, as observed in Bender *et al* (*13*), the $\delta Ar/N_2$-$\delta O_2/N_2$ slope (N.B. the independent and dependent variables here are different from the main text) is 0.46 in GISP2, 0.57 in Vostok 3G, 0.26 in Dome C (181-651 m), and 0.42 in Bryd ice core. The shared variance can be explained by the control on bubble close-off exerted by the ice properties modulated by summer insolation. A model developed by Kobashi *et al* (*22*) offers a quantitative estimate of the magnitude of the bubble close-off fractionations. This model shows that microbubbles control the magnitude of $\delta Ar/N_2$ and $\delta O_2/N_2$ fractionation within the firn with a $\delta Ar/N_2$-$\delta O_2/N_2$ slope of 0.55, consistent with the range of the observed slopes reported in Bender *et al* (*13*). The covariance between $\delta O_2/N_2$ and $\delta Ar/N_2$ originating from insolation modulation thus emerges as a potential way of deducing the true atmospheric O_2/N_2 ratio from the ice core $\delta O_2/N_2$ and $\delta Ar/N_2$, where $\delta Ar/N_2$ can be seen as a proxy for insolation.

However, the use of $\delta Ar/N_2$ to correct $\delta O_2/N_2$ for bubble close-off fractionations is complicated by another gas-fractionating process: post-coring gas loss. Gas losses further lower the ice core $\delta O_2/N_2$ and $\delta Ar/N_2$ and enriches $\delta^{18}O$ of O_2. This effect becomes apparent during ice storage at > −50 °C (*57*, *74*), when the ice has many fractures in it (*23*), and if the ice is subject to prolonged pumping [e.g. Allan Hills blue ice $\delta O_2/N_2$ data reported in Higgins *et al* (*53*)]. The temperature-dependency is consistent with a physical model that attributes gas loss to the outward diffusion of gases trapped inside the ice (*75*, *76*). Intriguingly, gas loss fractionation is still associated with an apparent ~0.5 slope of $\delta Ar/N_2$ against $\delta O_2/N_2$ (*23*), which is not very different from the slope caused by bubble close-off fractionations. Two modes of gas loss fractionation—one mass-dependent and the other size-dependent—are invoked to explain the observed slope as well as clearly mass-dependent isotopic fractionation (*22*, *23*). The slow leakage of gas molecules through the ice lattice is size-dependent and has little fractionation to the isotope ratios, while rapid gas loss associated with core cracking in response to rapid depressurization (and similarly to pumping) is mass dependent (*23*). The varying degree of each proposed process could cause scatters in the paired $\delta O_2/N_2$-$\delta Ar/N_2$ data but we cannot quantify their relative contribution. However, if gas loss fractionates $\delta O_2/N_2$ and $\delta Ar/N_2$ quantitatively similar to what bubble close-off does, $\delta Ar/N_2$ may still be capable of correcting $\delta O_2/N_2$ in a lump-sum way.

Below, we evaluate the effect of gas losses on O_2/N_2 and Ar/N_2 ratios preserved in the Allan Hills ice (batch 2 and 3 only) by plotting the difference between $\delta O_2/N_2$ replicates ($\Delta\delta O_2/N_2$) against the difference between $\delta Ar/N_2$ replicates ($\Delta\delta Ar/N_2$) measured on the same depth (Supplementary Data Table 2; Fig. S8). This difference should reflect the varying degree of gas losses experienced by each replicate plus the analytical uncertainties associated with gas handling and mass spec analyses. In the case where more than 2 replicates are available, the pair with the largest $\delta O_2/N_2$ difference is selected. The replicate with higher

$\delta O_2/N_2$ does not necessarily experience no gas loss. That sample simply has the least degree of gas losses. As a matter of fact, we have no way of identifying the $\delta O_2/N_2$ or $\delta Ar/N_2$ value that is truly "gas loss-free" for blue ice. At any rate, a slope of 1.47 ± 0.15 (2σ) is found between $\Delta\delta O_2/N_2$ and $\Delta\delta Ar/N_2$ in the Allan Hills blue ice (Fig. S8), close to the slope observed in the $\delta O_2/N_2$-$\delta Ar/N_2$ plot (Table 1). The correction by $\delta Ar/N_2$ would therefore also compensate the influence of post-coring gas loss on $\delta O_2/N_2$, in addition to bubble close-off fractionations. When $\delta Ar/N_2$ is corrected to a value smaller than 0 (e.g. the mean value of all observed $\delta Ar/N_2$), the corrected $\delta O_2/N_2$ is expected to have the same degree of gas loss and a constant offset from the true atmospheric $\delta O_2/N_2$ (Fig. 1). When the $\delta Ar/N_2$ is normalized to 0, the effect of gas loss is fully removed. We opted to apply the first type of correction to remove the imprint of insolation in $\delta O_2/N_2$ while retaining a (presumed) constant offset. The offset was subsequently accounted for by the *a priori* knowledge that the extrapolated $\delta O_2/N_2$ should be 0 at present (Fig. 4) (*8*). The approach adopted here has smaller uncertainties than extrapolating $\delta Ar/N_2$ to 0 does.

In sum, despite a lack of definitive knowledge on how insolation modulates $\delta O_2/N_2$ and $\delta Ar/N_2$ and how ice physics dictates post-coring gas loss fractionation, the prevalent, consistent, and strong correlation between ice core $\delta O_2/N_2$ and $\delta Ar/N_2$ creates an opportunity to use $\delta Ar/N_2$ for empirical $\delta O_2/N_2$ corrections and infer atmospheric pO_2 from the ice core $\delta O_2/N_2$.

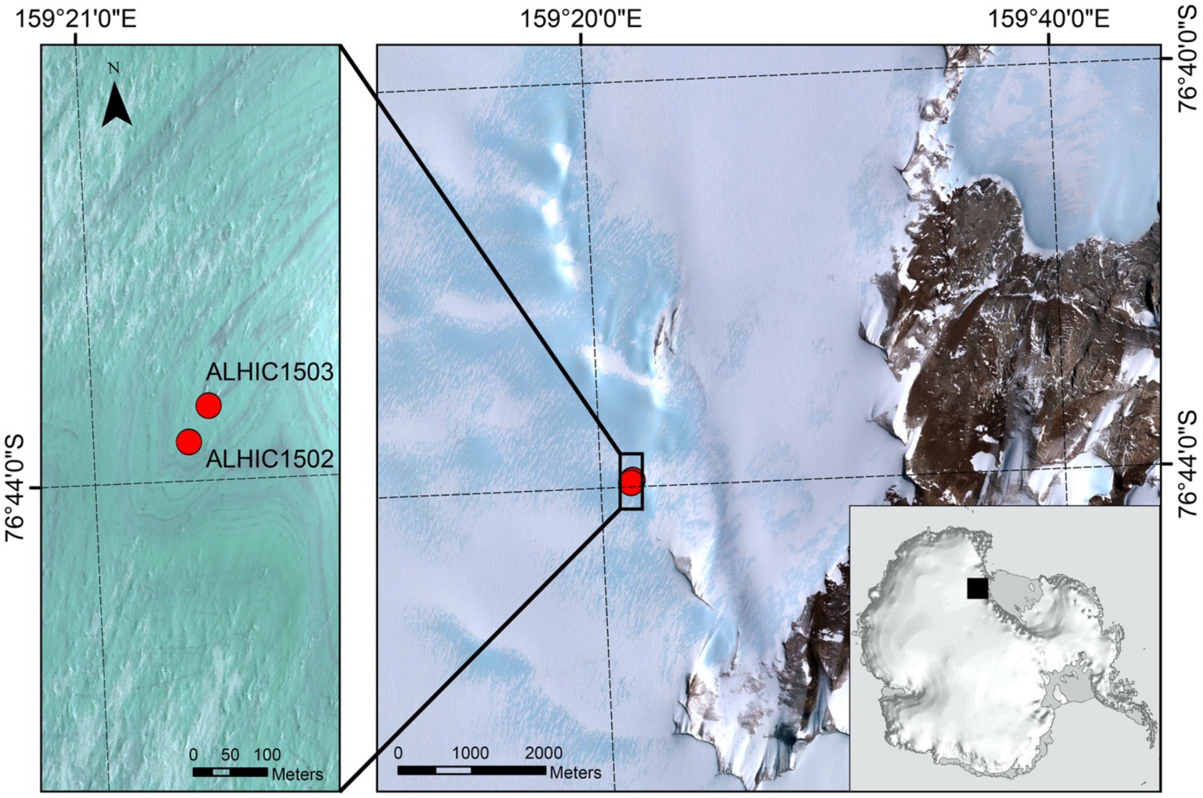

Fig. S1. Satellite imagery of the Allan Hills study area showing the locations of ALHIC1502 and ALHIC1503 boreholes. Right, WorldView03 true-color pan-sharpened imagery (copyright 2011, DigitalGlobe, Inc.) of the main ice field. The black outcrop in the main image is the Allan Hills nunatak. Inset, Antarctica with hill shading (source: Australian Antarctic Data Centre, Map 13469; licensed under a Creative Commons Attribution 3.0 Unported License). The study area is indicated by the black square. Left, a magnified image of the drilling site from same WorldView03 satellite file. The imagery is processed (gamma-adjusted) on ESRI® ArcGIS software package to enhance the color contrast within the blue ice. This color rendition causes blue ice to exhibit a green hue. The brown contours in the ice are exposed dust bands, providing a first-order tracer of surface ice stratigraphy. The locations of the cores reported in this work (see text) are marked with red circles.

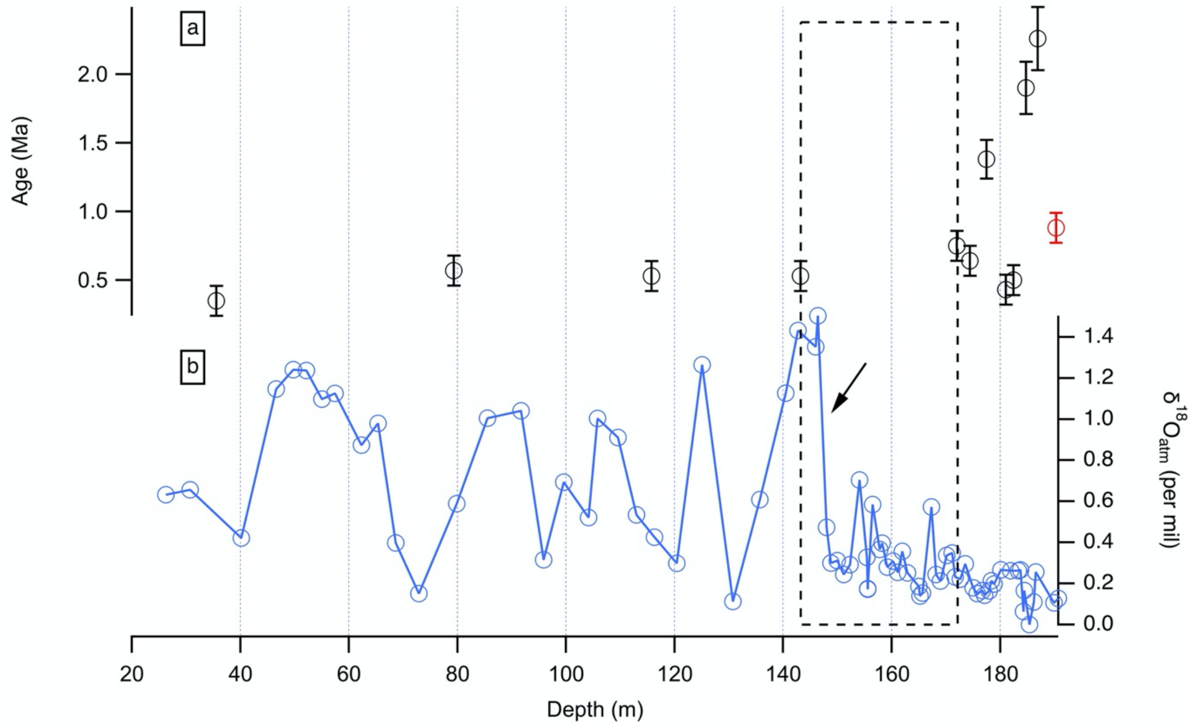

Fig. S2. (a) $^{40}Ar_{atm}$-derived ages and (b) $\delta^{18}O$ of O_2 ($\delta^{18}O_{atm}$) in the trapped air in ALHIC1502. Due to the sparse $^{40}Ar_{atm}$ data, the transition from post-MPT to MPT ice may occur between 143.19 and 172 m (shown as the dashed rectangle). We use $\delta^{18}O_{atm}$ as a stratigraphic marker to better identify the transition. In ALHIC1503, the first appearance of MPT ice is accompanied by a rapid decline of $\delta^{18}O_{atm}$ from 1.361‰ to 0.488‰. In ALHIC1502, this transition occurs between 146.40 and 147.99 m (marked by the black arrow). We therefore choose the average depth of 147.20 m as the divide between post-MPT and MPT ice. The deepest $^{40}Ar_{atm}$ datum marked in red was excluded from age classifications.

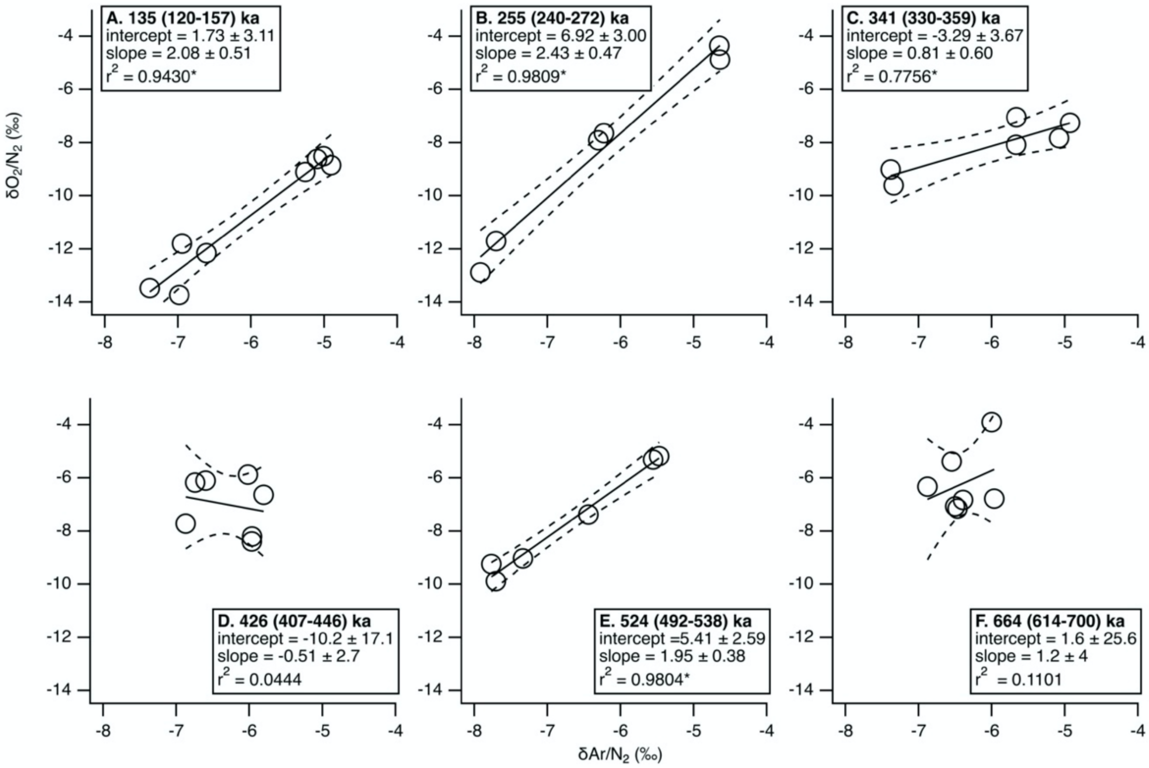

Fig. S3. Applying δAr/N_2-correction to EDC δO_2/N_2 data reported in Haeberli *et al* (*25*). Six age bins were selected as (A)-(F), named after the average age of all samples in each bin with the full age range shown in brackets. Linear regression results (intercepts and slopes with 95% CI) are also shown. Statistically significant correlations were marked with asterisks after the correlation coefficients. The poor correlation in (D) and (F) likely results from the small range of the observed δAr/N_2, leading to large uncertainties in the estimated δO_2/N_2 at 426 ka and 664 ka, as shown in Fig. 2. However, despite the large errors, the corrected δO_2/N_2 in (D) and (F) still falls on the trend line of the composite δO_2/N_2 record within the 95% CI.

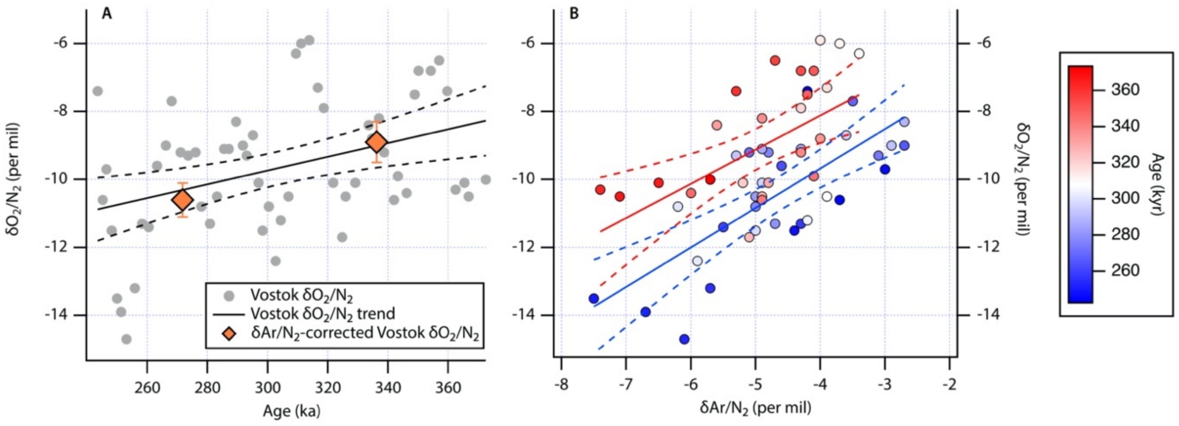

$\delta O_2/N_2 = (1.16 \pm 0.44) * \delta Ar/N_2 + (-5.03 \pm 2.05)$; r = 0.7393 (samples <300 ka)
$\delta O_2/N_2 = (1.00 \pm 0.59) * \delta Ar/N_2 + (-4.10 \pm 2.98)$; r = 0.5553 (samples >300 ka)

Fig. S4. $\delta O_2/N_2$ and $\delta Ar/N_2$ data between 243.5 and 372.5 ka recorded in Vostok ice core. (**A**) $\delta O_2/N_2$ plotted against age. The solid line indicates the trend of the Vostok $\delta O_2/N_2$ over time [−20.18 ± 6.53 ‰/Myr (95% CI)]. This trend is reproduced by corrected $\delta O_2/N_2$ data (orange squares with error bars representing the 95% confidence interval). (**B**) Paired $\delta O_2/N_2$-$\delta Ar/N_2$ data color-coded according to age. Older samples generally have higher $\delta O_2/N_2$ values compared to younger samples and qualitatively reflect the pO_2 decline in the Late-Pleistocene. The red and blue lines are regressions lines of $\delta O_2/N_2$ against $\delta Ar/N_2$ for samples older and younger than 300 ka, respectively, the results of which are shown in the box. The inferred rate of change solely based on these two regression lines is −26.00 ± 12.37 ‰/Myr (95% CI).

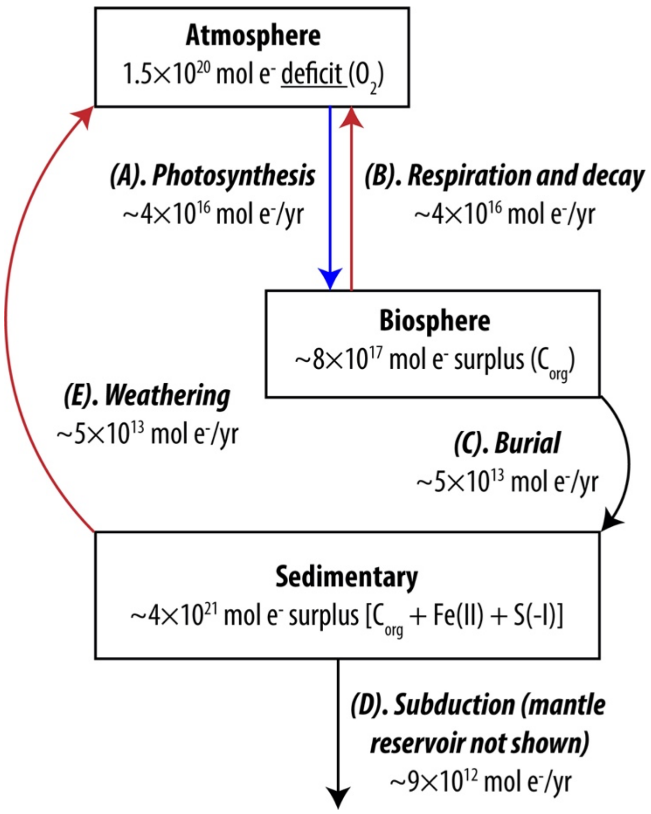

Fig. S5. A simplified view on the geochemical cycling of oxygen, represented by electron transfers. Assuming that O(-II), C(IV), Fe(III), and S(VI) are the most energetically favored redox states, deficit and surplus of electrons represent molecular O_2 and reduced carbon, iron, and sulfur species, respectively. Arrows represent the flow of electrons (red: consuming O_2; blue: producing O_2). For example, the generation of 1 mol of O_2 during photosynthesis means the transfer of 4 mol of electrons from O(-II) in water to another electron acceptor. Carbon, iron, and sulfur are common electron acceptors and the internal electron transfer between them (e.g. sulfate reduction) do not impact the oxygen budget. Numbers are estimates from Johnson and Bif (*77*) for photosynthesis and respiration rates, Galvez (*78*) for burial and weathering rates, and Walker (*79*) for reservoir sizes. Note that we do not differentiate igneous sink [e.g. Fe(II) upwelled through the mid-ocean ridges and volcanically outgassed H_2S] from the general weather sink, because the igneous sink flux is ~20% of the sedimentary weathering flux and consequently could only affect pO_2 on timescales beyond what ice core records can capture (*78*). These estimates all have their uncertainties, which are not discussed here because evaluating these estimates is beyond the scope of the present study.

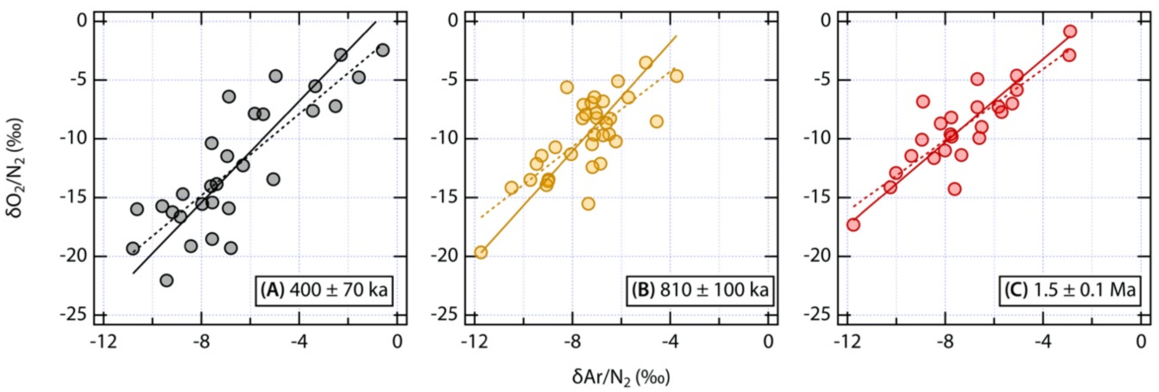

Fig. S6. Regression results using York Fit method (solid lines) and the OLS method (dashed lines) in three intervals described in *Materials and Methods*. Considering the analytical uncertainties (listed in Table S1) affects the estimates of slope and intercept. However, the estimated $\delta O_2/N_2$ is not very sensitive to the choice of regression methods when $\delta Ar/N_2$ is normalized to −7.1‰, where two regression lines intersect.

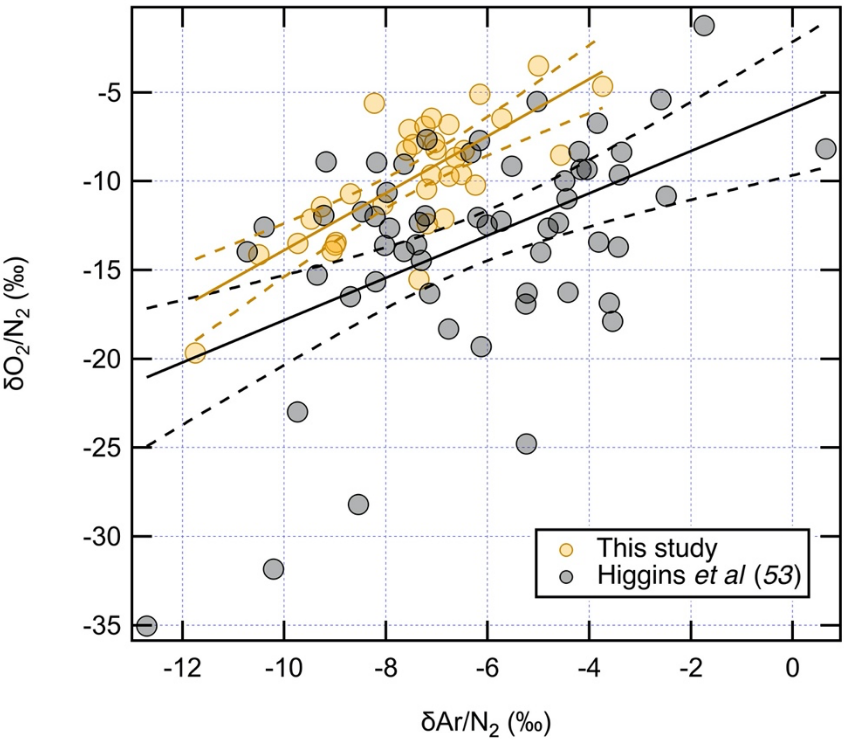

Fig. S7. MPT $\delta O_2/N_2$ versus $\delta Ar/N_2$ data measured in this study compared to Higgins *et al* (*53*) data. Data reported in Higgins *et al* (*53*) are associated with a wider spread in the data, a smaller $\delta O_2/N_2$-$\delta Ar/N_2$ slope with larger uncertainties [1.19 ± 0.56 (2σ)], and more negative $\delta O_2/N_2$ ratios.

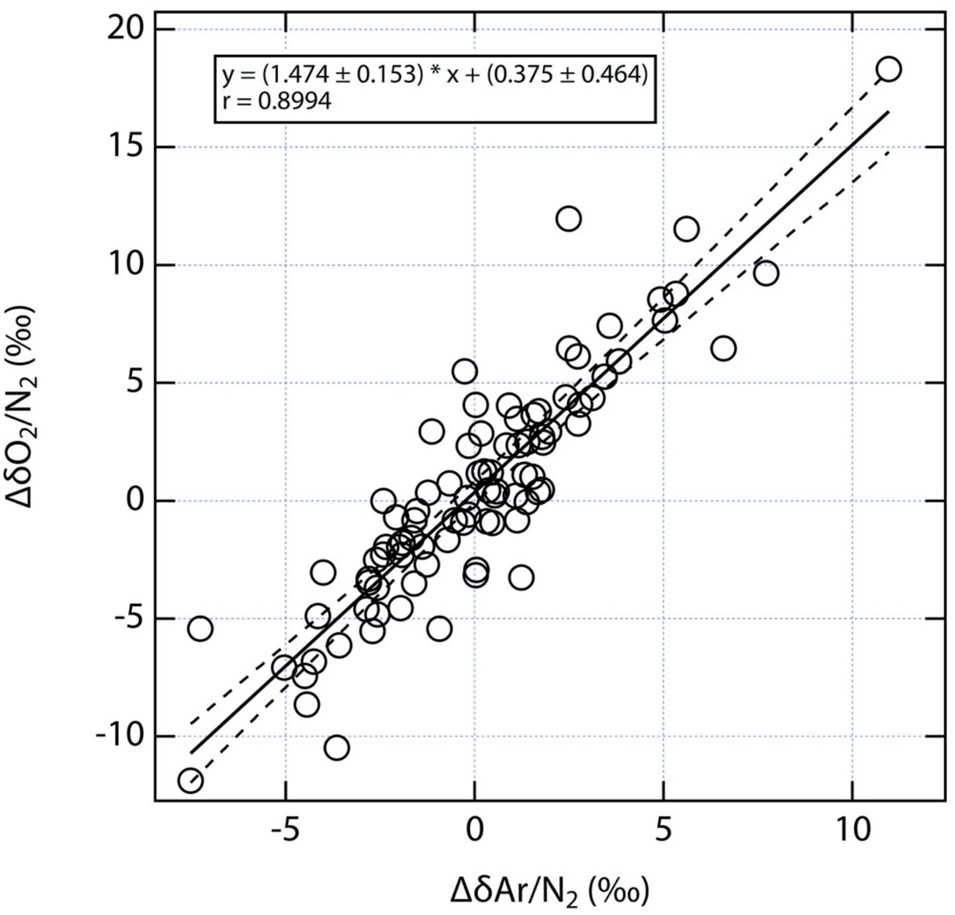

Fig. S8. Pair difference for measured $\delta O_2/N_2$ versus $\delta Ar/N_2$, calculated by subtracting the second-measured replicate from the first-measured replicate. For samples more than twice, we select two replicates with the largest $\delta O_2/N_2$ difference. The observed correlation is consistent with fractionation associated with post-coring gas losses in ice cores (*23*). Notably the slope of 1.474 ± 0.153 (2σ) is close to the observed slopes of $\delta O_2/N_2$ versus $\delta Ar/N_2$ in Allan Hills ice samples (Table 1).

Table S1. Comparing Ordinary-Least-Squares (OLS) linear regression of Allan Hills $\delta O_2/N_2$ against $\delta Ar/N_2$ with linear regression by York Fit (YF). Errors are given as 95% confidence intervals.

	post-MPT (400 ± 70 ka)	MPT (810 ± 100 ka)	pre-MPT (1.5 ± 0.1 Ma)
Slope OLS	1.74 ± 0.44	1.60 ± 0.50	1.51 ± 0.38
Slope YF	2.16 ± 0.84	2.31 ± 1.40	1.77 ± 1.00
y-intercept OLS	−0.9 ± 3.1‰	2.2± 3.8‰	2.0 ± 2.9‰
y-intercept YF	1.9 ± 5.9‰	7.4 ± 10.6‰	3.9 ± 7.5‰
Corrected $\delta O_2/N_2$ OLS (when δAr/N2 = −7.1‰)	−13.3 ± 1.2‰	−9.2 ± 0.8‰	−8.7 ± 0.8‰
Corrected $\delta O_2/N_2$ YF (when δAr/N2 = −7.1‰)	−13.5 ± 1.7‰	−9.0 ± 1.0‰	−8.7 ± 1.1‰

Data S1. Argon isotope composition in Allan Hills ice cores (separate file)

Data S2. Elemental and isotopic composition of nitrogen, oxygen, and argon in Allan Hills ice cores (separate file)

REFERENCES AND NOTES

1. R. A. Berner, Phanerozoic atmospheric oxygen: New results using the GEOCARBSULF model. *Am. J. Sci.* **309**, 603–606 (2009).

2. P. G. Falkowski, M. E. Katz, A. J. Milligan, K. Fennel, B. S. Cramer, M. P. Aubry, R. A. Berner, M. J. Novacek, W. M. Zapol, The rise of oxygen over the past 205 million years and the evolution of large placental mammals. *Science* **309**, 2202–2204 (2005).

3. N. J. Shackleton, The carbon isotope record of the Cenozoic: History of organic carbon burial and of oxygen in the ocean and atmosphere. *Geol. Soc. Lond. Spec. Publ.* **26**, 423–434 (1987).

4. J. Farquhar, H. Bao, M. Thiemens, Atmospheric influence of Earth's earliest sulfur cycle. *Science* **289**, 756–758 (2000).

5. E. J. Bellefroid, A. V. S. Hood, P. F. Hoffman, M. D. Thomas, C. T. Reinhard, N. J. Planavsky, Constraints on Paleoproterozoic atmospheric oxygen levels. *Proc. Natl. Acad. Sci. U.S.A.* **115**, 8104–8109 (2018).

6. D. S. Hardisty, Z. Lu, N. J. Planavsky, A. Bekker, P. Philippot, X. Zhou, T. W. Lyons, An iodine record of paleoproterozoic surface ocean oxygenation. *Geology* **42**, 619–622 (2014).

7. I. J. Glasspool, A. C. Scott, Phanerozoic concentrations of atmospheric oxygen reconstructed from sedimentary charcoal. *Nat. Geosci.* **3**, 627–630 (2010).

8. D. A. Stolper, M. L. Bender, G. B. Dreyfus, Y. Yan, J. A. Higgins, A Pleistocene ice core record of atmospheric O_2 concentrations. *Science* **353**, 1427–1430 (2016).

9. R. A. Berner, G. P. Landis, Gas bubbles in fossil amber as possible indicators of the major gas composition of ancient air. *Science* **239**, 1406–1409 (1988).

10. N. J. Blamey, U. Brand, J. Parnell, N. Spear, C. Lécuyer, K. Benison, F. Meng, P. Ni, Paradigm shift in determining Neoproterozoic atmospheric oxygen. *Geology* **44**, 651–654 (2016).

11. L. Y. Yeung, Low oxygen and argon in the Neoproterozoic atmosphere at 815 Ma. *Earth Planet. Sci. Lett.* **480**, 66–74 (2017).

12. T. Sowers, M. Bender, D. Raynaud, Elemental and isotopic composition of occluded O_2 and N_2 in polar ice. *J. Geophys. Res. Atmos.* **94**, 5137–5150 (1989).

13. M. Bender, T. Sowers, V. Lipenkov, On the concentrations of O_2, N_2, and Ar in trapped gases from ice cores. *J. Geophys. Res. Atmos.* **100**, 18651–18660 (1995).

14. A. Landais, G. Dreyfus, E. Capron, K. Pol, M.-F. Loutre, D. Raynaud, V. Y. Lipenkov, L. Arnaud, V. Masson-Delmotte, D. Paillard, Towards orbital dating of the EPICA Dome C ice core using $\delta O_2/N_2$. *Clim. Past* **8**, 191–203 (2012).

15. T. Extier, A. Landais, C. Bréant, F. Prié, L. Bazin, G. Dreyfus, D. M. Roche, M. Leuenberger, On the use of $\delta^{18}O_{atm}$ for ice core dating. *Quat. Sci. Rev.* **185**, 244–257 (2018).

16. R. A. Berner, Geological nitrogen cycle and atmospheric N_2 over Phanerozoic time. *Geology* **34**, 413–415 (2006).

17. F. Herman, J.-D. Champagnac, Plio-Pleistocene increase of erosion rates in mountain belts in response to climate change. *Terra Nova* **28**, 2–10 (2016).

18. M. Kölling, I. Bouimetarhan, M. W. Bowles, T. Felis, T. Goldhammer, K.-U. Hinrichs, M. Schulz, M. Zabel, Consistent CO_2 release by pyrite oxidation on continental shelves prior to glacial terminations. *Nat. Geosci.* **12**, 929–934 (2019).

19. Y. Yan, M. L. Bender, E. J. Brook, H. M. Clifford, P. C. Kemeny, A. V. Kurbatov, S. Mackay, P. A. Mayewski, J. Ng, J. P. Severinghaus, J. A. Higgins, Two-million-year-old snapshots of atmospheric gases from Antarctic ice. *Nature* **574**, 663–666 (2019).

20. I. M. Whillans, W. A. Cassidy, Catch a falling star: Meteorites and old ice. *Science* **222**, 55–57 (1983).

21. M. L. Bender, B. Barnett, G. Dreyfus, J. Jouzel, D. Porcelli, The contemporary degassing rate of ^{40}Ar from the solid Earth. *Proc. Natl. Acad. Sci. U.S.A.* **105**, 8232–8237 (2008).

22. T. Kobashi, T. Ikeda-Fukazawa, M. Suwa, J. Schwander, T. Kameda, J. Lundin, A. Hori, H. Motoyama, M. Döring, M. Leuenberger, Post-bubble close-off fractionation of gases in polar firn and ice cores: Effects of accumulation rate on permeation through overloading pressure. *Atmos. Chem. Phys.* **15**, 13895–13914 (2015).

23. J. P. Severinghaus, R. Beaudette, M. A. Headly, K. Taylor, E. J. Brook, Oxygen-18 of O_2 records the impact of abrupt climate change on the terrestrial biosphere. *Science* **324**, 1431–1434 (2009).

24. M. L. Bender, Orbital tuning chronology for the Vostok climate record supported by trapped gas composition. *Earth Planet. Sci. Lett.* **204**, 275–289 (2002).

25. M. Haeberli, D. Baggenstos, J. Schmitt, M. Grimmer, A. Michel, T. Kellerhals, H. Fischer, Snapshots of mean ocean temperature over the last 700 000 years using noble gases in the EPICA Dome C ice core. *Clim. Past* **17**, 843–867 (2021).

26. H. Craig, Y. Horibe, T. Sowers, Gravitational separation of gases and isotopes in polar ice caps. *Science* **242**, 1675–1678 (1988).

27. H. D. Holland, Volcanic gases, black smokers, and the Great Oxidation Event. *Geochim. Cosmochim. Acta* **66**, 3811–3826 (2002).

28. A. Jeltsch-Thömmes, G. Battaglia, O. Cartapanis, S. L. Jaccard, F. Joos, Low terrestrial carbon storage at the Last Glacial Maximum: Constraints from multi-proxy data. *Clim. Past* **15**, 849–879 (2019).

29. E. A. G. Schuur, A. D. McGuire, C. Schädel, G. Grosse, J. W. Harden, D. J. Hayes, G. Hugelius, C. D. Koven, P. Kuhry, D. M. Lawrence, S. M. Natali, D. Olefeldt, V. E. Romanovsky, K. Schaefer, M. R. Turetsky, C. C. Treat, J. E. Vonk, Climate change and the permafrost carbon feedback. *Nature* **520**, 171–179 (2015).

30. L. E. Lisiecki, M. E. Raymo, A Pliocene-Pleistocene stack of 57 globally distributed benthic $\delta^{18}O$ records. *Paleoceanography* **20**, PA1003 (2005).

31. P. U. Clark, D. Archer, D. Pollard, J. D. Blum, J. A. Rial, V. Brovkin, A. C. Mix, N. G. Pisias, M. Roy, The middle Pleistocene transition: Characteristics. Mechanisms, and implications for long-term changes in atmospheric pCO_2. *Quat. Sci. Rev.* **25**, 3150–3184 (2006).

32. C. J. Berends, P. Köhler, L. J. Lourens, R. S. W. van de Wal, On the cause of the mid-pleistocene transition. *Rev. Geophys.* **59**, e2020RG000727 (2021).

33. H. L. Ford, M. E. Raymo, Regional and global signals in seawater $\delta^{18}O$ records across the mid-Pleistocene transition. *Geology* **48**, 113–117 (2019).

34. E. J. Rohling, G. L. Foster, K. M. Grant, G. Marino, A. P. Roberts, M. E. Tamisiea, F. Williams, Sea-level and deep-sea-temperature variability over the past 5.3 million years. *Nature* **508**, 477–482 (2014).

35. J. Boellstorff, North American Pleistocene stages reconsidered in light of probable pliocene-pleistocene continental glaciation. *Science* **202**, 305–307 (1978).

36. J. Ehlers, P. L. Gibbard, The extent and chronology of Cenozoic global glaciation. *Quat. Int.* **164-165**, 6–20 (2007).

37. S. Sosdian, Y. Rosenthal, Deep-sea temperature and ice volume changes across the Pliocene-Pleistocene climate transitions. *Science* **325**, 306–310 (2009).

38. T. B. Chalk, M. P. Hain, G. L. Foster, E. J. Rohling, P. F. Sexton, M. P. S. Badger, S. G. Cherry, A. P. Hasenfratz, G. H. Haug, S. L. Jaccard, A. Martinez-Garcia, H. Palike, R. D. Pancost, P. A. Wilson, Causes of ice age intensification across the mid-Pleistocene transition. *Proc. Natl. Acad. Sci. U.S.A.* **114**, 13114–13119 (2017).

39. S. P. S. Gulick, J. M. Jaeger, A. C. Mix, H. Asahi, H. Bahlburg, C. L. Belanger, G. B. B. Berbel, L. Childress, E. Cowan, L. Drab, M. Forwick, A. Fukumura, S. Ge, S. Gupta, A. Kioka, S. Konno, L. J. Le Vay, C. März, K. M. Matsuzaki, Erin L. Mc Clymont, C. Moy, J. Müller, A. Nakamura, T. Ojima,

F. R. Ribeiro, K. D. Ridgway, O. E. Romero, A. L. Slagle, J. S. Stoner, G. St-Onge, I. Suto, M. D. Walczak, L. L. Worthington, I. Bailey, E. Enkelmann, R. Reece, J. M. Swartz, Mid-Pleistocene climate transition drives net mass loss from rapidly uplifting St. Elias Mountains, Alaska. *Proc. Natl. Acad. Sci. U.S.A.* **112**, 15042–15047 (2015).

40. M. A. Torres, N. Moosdorf, J. Hartmann, J. F. Adkins, A. J. West, Glacial weathering, sulfide oxidation, and global carbon cycle feedbacks. *Proc. Natl. Acad. Sci. U.S.A.* **114**, 8716–8721 (2017).

41. K. Horan, R. G. Hilton, D. Selby, C. J. Ottley, D. R. Gröcke, M. Hicks, K. W. Burton, Mountain glaciation drives rapid oxidation of rock-bound organic carbon. *Sci. Adv.* **3**, e1701107 (2017).

42. A. S. Colman, F. T. Mackenzie, H. D. Holland, P. Van Cappellen, E. D. Ingall, Redox stabilization of the atmosphere and oceans and marine productivity. *Science* **275**, 406–408 (1997).

43. J. R. Farmer, B. Hönisch, L. L. Haynes, D. Kroon, S. Jung, H. L. Ford, M. E. Raymo, M. Jaume-Seguí, D. B. Bell, S. L. Goldstein, L. D. Pena, M. Yehudai, J. Kim, Deep Atlantic Ocean carbon storage and the rise of 100,000-year glacial cycles. *Nat. Geosci.* **12**, 355–360 (2019).

44. A. Jeltsch-Thömmes, F. Joos, Modeling the evolution of pulse-like perturbations in atmospheric carbon and carbon isotopes: The role of weathering–sedimentation imbalances. *Clim. Past* **16**, 423–451 (2020).

45. L. D. Pena, S. L. Goldstein, Thermohaline circulation crisis and impacts during the mid-Pleistocene transition. *Science* **345**, 318–322 (2014).

46. G. Delisle, J. Sievers, Sub-Ice topography and meteorite finds near the Allan Hills and the near Western Ice Field, Victoria Land, Antarctica. *J. Geophys. Res. Planet.* **96**, 15577–15587 (1991).

47. L. Schultz, J. O. Annexstad, G. Delisle, Ice movement and mass balance at the Allan Hills Icefield. *Antarct. J. US* **25**, 94–95 (1990).

48. N. E. Spaulding, V. B. Spikes, G. S. Hamilton, P. A. Mayewski, N. W. Dunbar, R. P. Harvey, J. Schutt, A. V. Kurbatov, Ice motion and mass balance at the Allan Hills blue-ice area, Antarctica, with implications for paleoclimate reconstructions. *J. Glaciol.* **58**, 399–406 (2012).

49. R. Dadic, M. Schneebeli, N. A. N. Bertler, M. Schwikowski, M. Matzl, Extreme snow metamorphism in the Allan Hills, Antarctica, as an analogue for glacial conditions with implications for stable isotope composition. *J. Glaciol.* **61**, 1171–1182 (2015).

50. R. Bintanja, On the glaciological, meteorological, and climatological significance of Antarctic blue ice areas. *Rev. Geophys.* **37**, 337–359 (1999).

51. L. Kehrl, H. Conway, N. Holschuh, S. Campbell, A. V. Kurbatov, N. E. Spaulding, Evaluating the duration and continuity of potential climate records from the Allan Hills Blue Ice Area, East Antarctica. *Geophys. Res. Lett.* **45**, 4096–4104 (2018).

52. J. A. Menking, E. J. Brook, S. A. Shackleton, J. P. Severinghaus, M. N. Dyonisius, V. Petrenko, J. R. McConnell, R. H. Rhodes, T. K. Bauska, D. Baggenstos, Spatial pattern of accumulation at Taylor Dome during Marine Isotope Stage 4: Stratigraphic constraints from Taylor Glacier. *Clim. Past* **15**, 1537–1556 (2019).

53. J. A. Higgins, A. V. Kurbatov, N. E. Spaulding, E. Brook, D. S. Introne, L. M. Chimiak, Y. Z. Yan, P. A. Mayewski, M. L. Bender, Atmospheric composition 1 million years ago from blue ice in the Allan Hills, Antarctica. *Proc. Natl. Acad. Sci. U.S.A.* **112**, 6887–6891 (2015).

54. G. B. Dreyfus, F. Parrenin, B. Lemieux-Dudon, G. Durand, V. Masson-Delmotte, J. Jouzel, J. M. Barnola, L. Panno, R. Spahni, A. Tisserand, U. Siegenthaler, M. Leuenberger, Anomalous flow below 2700 m in the EPICA Dome C ice core detected using $\delta^{18}O$ of atmospheric oxygen measurements. *Clim. Past* **3**, 341–353 (2007).

55. S. Emerson, P. D. Quay, C. Stump, D. Wilbur, R. Schudlich, Chemical tracers of productivity and respiration in the subtropical Pacific Ocean. *J. Geophys. Res. Oceans* **100**, 15873–15887 (1995).

56. K. Kawamura, F. Parrenin, L. Lisiecki, R. Uemura, F. Vimeux, J. P. Severinghaus, M. A. Hutterli, T. Nakazawa, S. Aoki, J. Jouzel, M. E. Raymo, K. Matsumoto, H. Nakata, H. Motoyama, S. Fujita, K. Goto-Azuma, Y. Fujii, O. Watanabe, Northern Hemisphere forcing of climatic cycles in Antarctica over the past 360,000 years. *Nature* **448**, 912–916 (2007).

57. M. Suwa, M. L. Bender, Chronology of the Vostok ice core constrained by O_2/N_2 ratios of occluded air, and its implication for the Vostok climate records. *Quat. Sci. Rev.* **27**, 1093–1106 (2008).

58. D. York, N. M. Evensen, M. L. Martınez, J. De Basabe Delgado, Unified equations for the slope, intercept, and standard errors of the best straight line. *Am. J. Phys.* **72**, 367–375 (2004).

59. G. Baccolo, B. Delmonte, P. B. Niles, G. Cibin, E. D. Stefano, D. Hampai, L. Keller, V. Maggi, A. Marcelli, J. Michalski, C. Snead, M. Frezzotti, Jarosite formation in deep Antarctic ice provides a window into acidic, water-limited weathering on Mars. *Nat. Commun.* **12**, 436 (2021).

60. S. M. Aarons, S. M. Aciego, P. Gabrielli, B. Delmonte, J. M. Koornneef, A. Wegner, M. A. Blakowski, The impact of glacier retreat from the Ross Sea on local climate: Characterization of mineral dust in the Taylor Dome ice core, East Antarctica. *Earth Planet. Sci. Lett.* **444**, 34–44 (2016).

61. B. Bereiter, H. Fischer, J. Schwander, T. F. Stocker, Diffusive equilibration of N_2, O_2 and CO_2 mixing ratios in a 1.5-million-years-old ice core. *The Cryosphere* **8**, 245–256 (2014).

62. J.-L Tison, M de Angelis, G. Littot, E. Wolff, H. Fischer, M. Hansson, M. Bigler, R. Udisti, A. Wegner, J. Jouzel, B. Stenni, S. Johnsen, V. Masson-Delmotte, A. Landais, V. Lipenkov, L. Loulergue, J.-M Barnola, J.-R Petit, B. Delmonte, G. Dreyfus, D Dahl-Jensen, G. Durand, B. Bereiter, A. Schilt, R. Spahni, K. Pol, R. Lorrain, R. Souchez, D. Samyn, Retrieving the paleoclimatic signal from the deeper part of the EPICA Dome C ice core. *The Cryosphere* **9**, 1633–1648 (2015).

63. K. Pol, V. Masson-Delmotte, S. Johnsen, M. Bigler, O. Cattani, G. Durand, S. Falourd, J. Jouzel, B. Minster, F. Parrenin, C. Ritz, H. C. Steen-Larsen, B. Stenni, New MIS 19 EPICA Dome C high resolution deuterium data: Hints for a problematic preservation of climate variability at sub-millennial scale in the "oldest ice". *Earth Planet. Sci. Lett.* **298**, 95–103 (2010).

64. J. R. Petit, J. Jouzel, D. Raynaud, N. I. Barkov, J. M. Barnola, I. Basile, M. Bender, J. Chappellaz, M. Davis, G. Delaygue, M. Delmotte, V. M. Kotlyakov, M. Legrand, V. Y. Lipenkov, C. Lorius, L. Pepin, C. Ritz, E. Saltzman, M. Stievenard, Climate and atmospheric history of the past 420,000 years from the Vostok ice core, Antarctica. *Nature* **399**, 429–436 (1999).

65. U. Siegenthaler, T. F. Stocker, E. Monnin, D. Luthi, J. Schwander, B. Stauffer, D. Raynaud, J. M. Barnola, H. Fischer, V. Masson-Delmotte, J. Jouzel, Stable carbon cycle-climate relationship during the late Pleistocene. *Science* **310**, 1313–1317 (2005).

66. D. Lüthi, M. Le Floch, B. Bereiter, T. Blunier, J. M. Barnola, U. Siegenthaler, D. Raynaud, J. Jouzel, H. Fischer, K. Kawamura, T. F. Stocker, High-resolution carbon dioxide concentration record 650,000-800,000 years before present. *Nature* **453**, 379–382 (2008).

67. B. Bereiter, S. Eggleston, J. Schmitt, C. Nehrbass-Ahles, T. F. Stocker, H. Fischer, S. Kipfstuhl, J. Chappellaz, Revision of the EPICA Dome C CO_2 record from 800 to 600 kyr before present. *Geophys. Res. Lett.* **42**, 542–549 (2015).

68. J. P. Severinghaus, A. Grachev, M. Battle, Thermal fractionation of air in polar firn by seasonal temperature gradients. *Geochem. Geophys. Geosyst.* **2**, 2000GC000146 (2001).

69. J. P. Severinghaus, M. O. Battle, Fractionation of gases in polar ice during bubble close-off: New constraints from firn air Ne, Kr and Xe observations. *Earth Planet. Sci. Lett.* **244**, 474–500 (2006).

70. M. M. Herron, C. C. Langway, Firn densification: An empirical model. *J. Glaciol.* **25**, 373–385 (1980).

71. C. Huber, U. Beyerle, M. Leuenberger, J. Schwander, R. Kipfer, R. Spahni, J. P. Severinghaus, K. Weiler, Evidence for molecular size dependent gas fractionation in firn air derived from noble gases, oxygen, and nitrogen measurements. *Earth Planet. Sci. Lett.* **243**, 61–73 (2006).

72. M. O. Battle, J. P. Severinghaus, E. D. Sofen, D. Plotkin, A. J. Orsi, M. Aydin, S. A. Montzka, T. Sowers, P. P. Tans, Controls on the movement and composition of firn air at the West Antarctic Ice Sheet Divide. *Atmos. Chem. Phys.* **11**, 11007–11021 (2011).

73. S. Fujita, J. Okuyama, A. Hori, T. Hondoh, Metamorphism of stratified firn at Dome Fuji, Antarctica: A mechanism for local insolation modulation of gas transport conditions during bubble close off. *J. Geophys. Res. Earth Surface* **114**, F03023 (2009).

74. A. Landais, J. Chappellaz, M. Delmotte, J. Jouzel, T. Blunier, C. Bourg, N. Caillon, S. Cherrier, B. Malaizé, V. Masson-Delmotte, D. Raynaud, J. Schwander, J. P. Steffensen, A tentative reconstruction of the last interglacial and glacial inception in Greenland based on new gas measurements in the Greenland Ice Core Project (GRIP) ice core. *J. Geophys. Res. Atmos.* **108**, 4563 (2003).

75. T. Ikeda-Fukazawa, K. Fukumizu, K. Kawamura, S. Aoki, T. Nakazawa, T. Hondoh, Effects of molecular diffusion on trapped gas composition in polar ice cores. *Earth Planet. Sci. Lett.* **229**, 183–192 (2005).

76. B. Bereiter, J. Schwander, D. Lüthi, T. F. Stocker, Change in CO_2 concentration and O_2/N_2 ratio in ice cores due to molecular diffusion. *Geophys. Res. Lett.* **36**, L05703 (2009).

77. K. S. Johnson, M. B. Bif, Constraint on net primary productivity of the global ocean by Argo oxygen measurements. *Nat. Geosci.* **14**, 769–774 (2021).

78. M. E. Galvez, Redox constraints on a Cenozoic imbalance in the organic carbon cycle. *Am. J. Sci.* **320**, 730–751 (2020).

79. J. C. Walker, Stability of atmospheric oxygen. *Am. J. Sci.* **274**, 193–214 (1974).
